# Plasmin activity promotes amyloid deposition in a transgenic model of human transthyretin amyloidosis

**DOI:** 10.1038/s41467-021-27416-z

**Published:** 2021-12-07

**Authors:** Ivana Slamova, Rozita Adib, Stephan Ellmerich, Michal R. Golos, Janet A. Gilbertson, Nicola Botcher, Diana Canetti, Graham W. Taylor, Nigel Rendell, Glenys A. Tennent, Guglielmo Verona, Riccardo Porcari, P. Patrizia Mangione, Julian D. Gillmore, Mark B. Pepys, Vittorio Bellotti, Philip N. Hawkins, Raya Al-Shawi, J. Paul Simons

**Affiliations:** 1 Centre for Amyloidosis and Acute Phase Proteins, Division of Medicine, University College London, London, UK; 2 Department of Molecular Medicine, Institute of Biochemistry, University of Pavia, Pavia, Italy; 3 Present Address: London Institute of Medical Sciences, Imperial College London, London, UK; 4 Present Address: School of Biological Sciences, University of Bristol, Bristol, UK

**Keywords:** Protein folding, Proteolysis, Cardiovascular biology, Experimental models of disease, Molecular medicine

## Abstract

Cardiac ATTR amyloidosis, a serious but much under-diagnosed form of cardiomyopathy, is caused by deposition of amyloid fibrils derived from the plasma protein transthyretin (TTR), but its pathogenesis is poorly understood and informative in vivo models have proved elusive. Here we report the generation of a mouse model of cardiac ATTR amyloidosis with transgenic expression of human TTR^S52P^. The model is characterised by substantial ATTR amyloid deposits in the heart and tongue. The amyloid fibrils contain both full-length human TTR protomers and the residue 49-127 cleavage fragment which are present in ATTR amyloidosis patients. Urokinase-type plasminogen activator (uPA) and plasmin are abundant within the cardiac and lingual amyloid deposits, which contain marked serine protease activity; knockout of α_2_-antiplasmin, the physiological inhibitor of plasmin, enhances amyloid formation. Together, these findings indicate that cardiac ATTR amyloid deposition involves local uPA-mediated generation of plasmin and cleavage of TTR, consistent with the previously described mechano-enzymatic hypothesis for cardiac ATTR amyloid formation. This experimental model of ATTR cardiomyopathy has potential to allow further investigations of the factors that influence human ATTR amyloid deposition and the development of new treatments.

## Abstract (web summary)

ATTR amyloidosis causes heart failure through the accumulation of misfolded transthyretin in cardiac muscle. Here the authors report a mouse model of ATTR amyloidosis and demonstrate the involvement of protease activity in ATTR amyloid deposition.

## Introduction

The systemic amyloidoses are a group of diseases in which globular proteins misfold and aggregate to form insoluble amyloid fibrils, which are deposited in the extracellular space where they disrupt tissue structure and organ function. Eighteen amyloid-forming proteins have been identified in human amyloidoses^1^, deposited in various and sometimes characteristic anatomical locations. One of these, transthyretin (TTR), is an abundant homotetrameric plasma protein which binds thyroxine and retinol binding protein. Deposits of ATTR amyloid derived from wild-type TTR are common in older individuals, most notably in the heart, but also the pulmonary vasculature, flexor retinaculum and ligamentum flavum^2–7^. The prevalence of ATTR amyloid deposits found at autopsy is ~25% in individuals beyond age 80 years^2,5^, but the frequency and spectrum of clinically significant disease caused by this type of amyloid is only beginning to become clear. Whilst minor deposits in an otherwise normal heart may merely be an incidental phenomenon, modest deposits may nevertheless contribute to cardiac dysfunction in combination with additional common pathologies such as aortic valve stenosis^8–12^. Until recently, substantial deposition of ATTR amyloid in the heart causing a symptomatic cardiomyopathy in its own right, causing intractable heart failure and life-threatening arrhythmias, was very rarely recognised. However, remarkable advances in cardiac magnetic resonance imaging^13^ and repurposing of radionuclide bone tracers that yield highly characteristic images^14,15^ now enable reliable non-invasive diagnosis^16^ of cardiac ATTR amyloidosis without recourse to heart biopsy, and have revealed that the prevalence of ATTR cardiomyopathy is substantial, especially in older men^17,18^. In contrast to these diagnostic advances, quality of life remains poor, survival short and development of treatment is in its infancy^17^.

Whereas wild-type ATTR amyloidosis is a disorder of older people, many point mutations in the TTR gene are associated with dominantly inherited susceptibility to forms of the disease that can develop at any time from early adult life onwards^19–23^. The most prevalent pathogenic allele, encoding TTR^V122I^, is carried by 3–4% of African American individuals, is widespread in Africa and black populations generally, and is associated with a much increased incidence of cardiac ATTR amyloidosis^24^. Most other amyloidogenic *TTR* mutations are associated with polyneuropathy in addition to cardiomyopathy, with variable visceral and other tissue involvement.

Key outstanding questions are the in vivo mechanisms of ATTR amyloid formation, including the basis for its characteristic deposition in certain anatomical locations and its marked male preponderance. A tractable experimental model of ATTR amyloidosis that closely reflects the human disease will enable these and other important phenomena to be investigated in vivo, as well as elucidating novel therapies.

Here, we report the generation of a mouse model of cardiac ATTR amyloidosis with transgenic expression of human TTR^S52P^. We selected this particular TTR variant following our clinical experience with a British family in whom this mutation was associated with the highly penetrant development of ATTR amyloidosis leading to death with cardiomyopathy during the third decade^25^. Many aspects of the reproducible formation of amyloid in this experimental model mirror key features of clinical ATTR amyloidosis, including the progressive accumulation of substantial amyloid deposits in the myocardium. The properties of the model provide strong support for the proposed mechano-enzymatic mechanism of ATTR amyloid formation^25–27^. Using the model, we confirm the dependence of amyloidogenesis on both fibril seeding and proteolytic cleavage of TTR, identify amyloid deposits as sites of abnormal proteolytic activity, and show that plasmin activity promotes ATTR amyloid deposition in vivo.

## Results

### Transgenic expression of TTR^S52P^ in mice

**Fig. 1:**
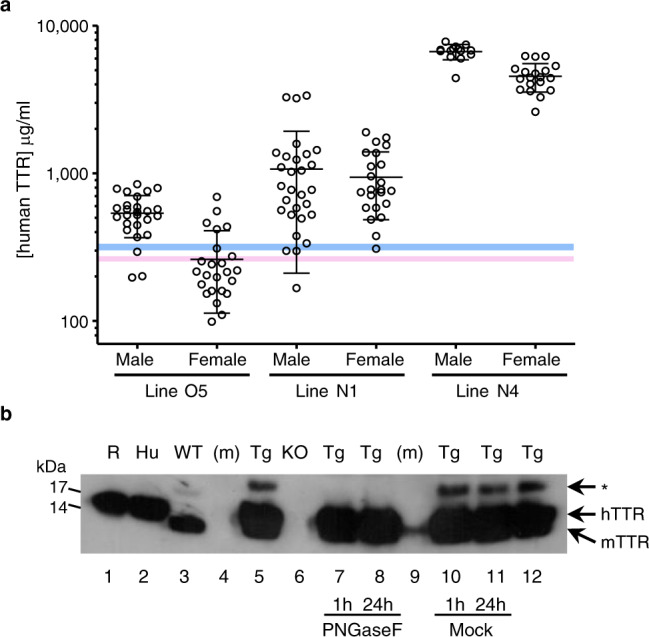
Human TTR protein in the circulation of *TTR*^S52P^ transgenic mice. **a** Human TTR concentrations in the sera of transgenic mice were measured by electroimmunoassay calibrated with isolated pure human wild-type TTR; non-transgenic mice gave no signal in this assay. Results (individual values, mean and SD) are presented for male and female transgenic mice of three independent lines that were also homozygous for a mouse *ttr* null allele (*n* = 26, 29 and 14 for male mice of lines O5, N1 and N4, respectively, and *n* = 24, 23 and 19 for female mice of lines O5, N1 and N4, respectively). The reported ranges of TTR concentrations in healthy adult women and men^64^ are indicated in pink and blue, respectively. **b** *N*-glycosylation of human TTR in serum of human *TTR*^S52P^ transgenic mice demonstrated by western blot probed with anti-human TTR antiserum. Transgenic mouse serum was treated with PNGaseF (lanes 7 & 8) under denaturing conditions for 1 or 24 h, as indicated; lanes 10 and 11 contained serum samples treated identically except for the omission of the PNGaseF enzyme, and lanes 5 and 12 contained untreated serum. The asterisk indicates the *N*-glycosylated human TTR^S52P^ protein. Other lanes contained recombinant human TTR (R), human serum (Hu), wild-type mouse serum (WT), mouse *ttr* knockout serum (KO) or molecular weight protein markers (m). The signal in lane 9 is a result of spillover from an adjacent lane. This experiment was not repeated. Source data are provided as a Source Data file.

We generated transgenic mice by pronuclear microinjection of a construct linking the human genomic sequence encoding the TTR^S52P^ variant to an albumin enhancer/promoter cassette^28^, to maximise expression of the human *TTR*^S52P^ transgene in hepatocytes. Three transgenic mouse lines expressing human TTR were established, named O5, N1 and N4. Mixed TTR tetramers containing protomers of both mouse and human TTR generated in vitro, or by co-expression in vivo, are more stable than native human TTR (refs. ^29,30^), which could potentially inhibit their potential to form amyloid. We therefore additionally bred the human *TTR*^S52P^ transgene onto the mouse *ttr* knockout background. The circulating concentrations of transgene-encoded human TTR in these lines of mice, after breeding onto the mouse *ttr* knockout background, ranged from being comparable to the normal human range in line O5, to >10-fold greater, in line N4 (Fig. 1a). A minor, reduced mobility band of human TTR was detected on prolonged exposure of western blots of sera from human TTR^S52P^ transgenic mice but was absent from control mouse and human sera. The band disappeared after PNGaseF treatment showing that it was *N*-glycosylated TTR (Fig. 1b), as previously reported for other amyloidogenic human TTR variants^31,32^. Native circulating TTR is not normally glycosylated. The atypical glycosylation targets misfolded intracellular TTR in the secretory pathway for degradation^31^ but some glycosylated TTR escapes into the circulation^32^. Its presence in our mice is consistent with the known marked instability of native human TTR^S52P^.

### Lack of spontaneous ATTR amyloid deposition

The tissues of a total of 59 human TTR^S52P^ transgenic mice taken at various ages were comprehensively studied by histochemical staining with alkaline alcoholic Congo red, viewed in intense cross polarised light. Amongst these mice, including 9 male and 9 female 22–26-month-old mice of line O5, and 6 male and 6 female 18–20-month-old mice of line N4, amyloid was present in only a single 24-month-old line O5 animal. However, the abundant deposits in the heart, liver, spleen and tongue of that animal were not ATTR amyloid, but were identified as mouse apolipoprotein AII (apoAII) amyloid both through immunohistochemical staining (Supplementary Fig. 1) and proteomic mass spectrometry. Thus despite the extremely amyloidogenic nature of human TTR^S52P^ in patients and in vitro, along with abundant transgenic expression, no spontaneous ATTR amyloid deposition was detected in any mouse.

### Seeded deposition of ATTR amyloid

**Fig. 2:**
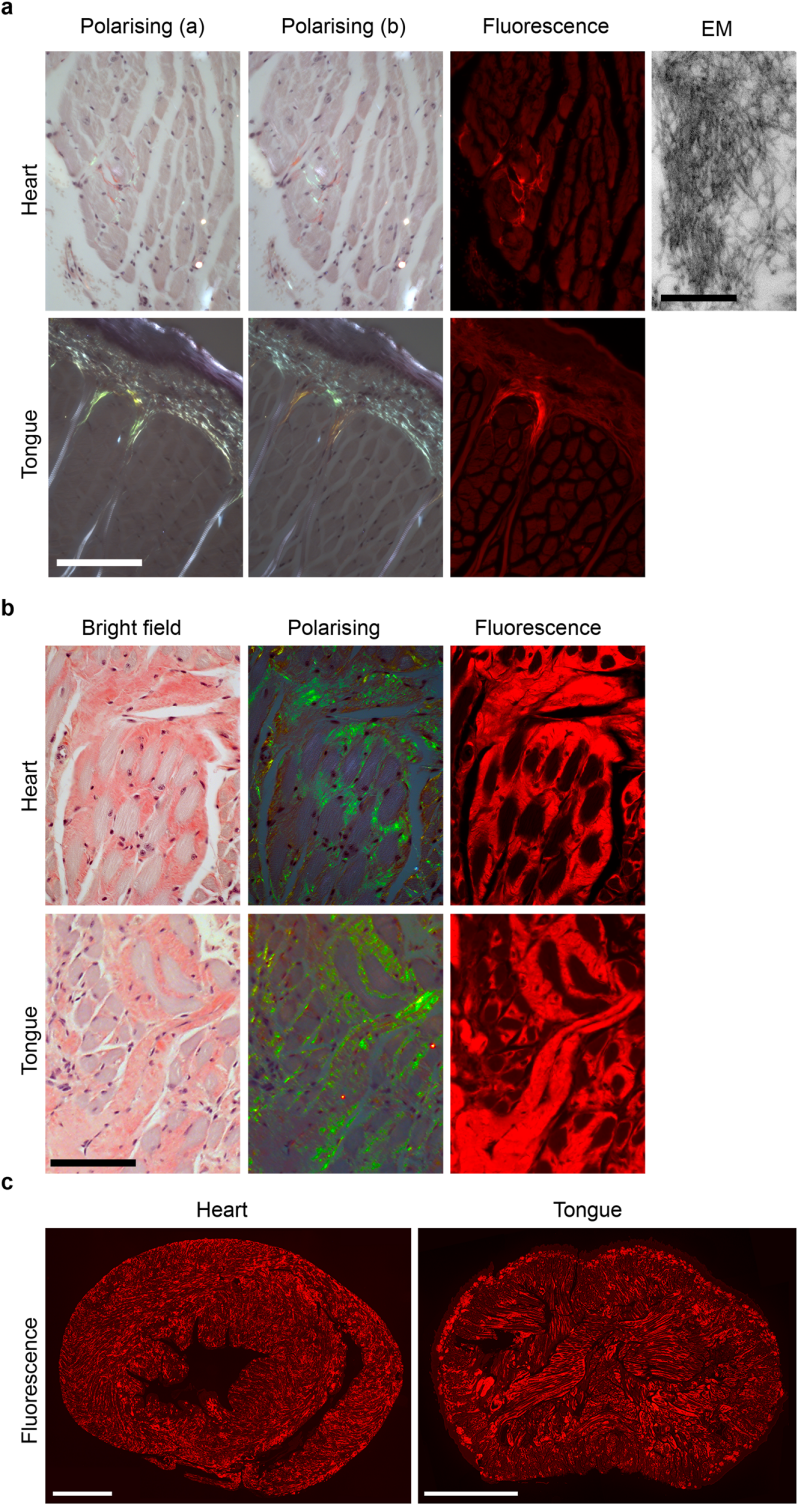
Amyloid in seeded mice of lines O5 and N4. Amyloid demonstrated by the pathognomonic red-green birefringence of Congo red stained sections viewed by polarising microscopy under intense illumination; background white birefringence was generated by myofibrils. The full extent of the amyloid is readily appreciated in the Congo red fluorescence images. **a** Minor but unequivocal amyloid deposits in the heart and tongue of a male line O5 mouse 7 months after seeding, representative of the maximum amyloid loads in mice of this line (*n* = 53 amyloidotic mice). The electron micrograph shows the unbranching ~10 nm diameter morphology characteristic of genuine amyloid fibrils. **b** Copious amyloid deposits in heart and tongue of a line N4 transgenic male mouse 6 months after seeding. **c** Low magnification Congo red fluorescence images showing the typical extent of amyloid deposits in heart and tongue of line N4 transgenic mice seeded 6 months prior to sample collection. **b**, **c** are representative of typical amyloid loads in mice of this line analysed 6-7 months after seeding (*n* = 10). Scale bars: **a**, 100 µm (light micrographs) and 500 nm (EM); **b**, 100 µm; **c**, 1 mm.

Seeding markedly promotes amyloid fibril formation in vitro and the augmenting role of minute amyloidotic organ extracts, so-called ‘amyloid enhancing factor’, in accelerating systemic AA amyloidosis in mice in vivo is well characterised^33^. To seed deposition of ATTR amyloid, we administered by intravenous injection to *TTR*^S52P^ transgenic mice tiny quantities of amyloid extracted from the spleen of a patient with ATTR^S52P^ amyloidosis. At 4–11 months thereafter, 39 of 51 treated line O5 mice had isolated, small, but unequivocal, amyloid deposits in the heart and/or tongue (Fig. 2a and supplementary table 1), though no amyloid was detected in any other site. The amyloid deposits in the transgenic mice were extracellular, located mostly between the muscle fibres and adjacent to blood vessels, and typically were greater in the tongue than in the heart. Consistent with the identification of amyloid by pathognomonic Congo red staining, extracellular deposits with the characteristic ultrastructural features of amyloid fibrils were confirmed by electron microscopy, demonstrating unbranched fibrils of ~10 nm diameter and indeterminate length (Fig. 2a).

To exclude the possibility that the minuscule quantities of administered ex vivo amyloid material could confound the findings, non-transgenic control mice receiving the identical injection of ATTR^S52P^ amyloid were analysed. No Congophilic amyloid was detected in the tissues of any of 22 mice at times ranging from 2 weeks to 7 months after injection. Furthermore, no human TTR was detected in the hearts, tongues, livers or spleens of two mice analysed 2 weeks after injection by western blotting, even with over-exposure, under conditions which readily detect TTR in ATTR amyloidotic tissues. These data show that the amyloid observed in the transgenic mice was newly deposited and not an artefact resulting from the injection of ATTR amyloid seeds.

Seeding of line N4 mice with patient-derived ATTR^S52P^ amyloid resulted in more rapid and far more extensive amyloid deposition than observed in line O5 mice (Fig. 2b and Supplementary Fig. 2), as anticipated because of the >10-fold greater circulating concentration of human TTR^S52P^ in the N4 line. One month after injection of ATTR^S52P^ amyloid fibrils, two of five seeded mice had positive amyloid histology and, after an additional month, all seeded mice analysed contained amyloid in heart and tongue (*n* = 20), typically in small, isolated patches. The amounts and distribution of amyloid deposition in heart and tongue increased progressively over time (Supplementary Fig. 2). The histological appearance of mouse and human ATTR^S52P^ cardiac amyloid were closely similar (Supplementary Fig. 3), and the deposits contained proteoglycans and serum amyloid P component (SAP), as expected (Supplementary Fig. 4). Amyloid deposits were also found in numerous other sites (Supplementary Figs. 5–7), including skeletal muscle, all regions of the gastrointestinal tract, thyroid glands and, in small amounts, in subcutaneous and visceral fat. Amyloid was frequently observed in nerve bundles within the tongues of seeded mice from 6 months after seeding. By contrast, in sciatic nerves, while amyloid was present in the epineurium, it was almost completely absent from within nerve bundles, even up to 12 months after seeding. Specks of amyloid were detectable in spleens and kidneys from 6 months after seeding, though the amounts of amyloid in these organs remained very small even 12 months after seeding. The amyloid deposits were not associated with local inflammation, and amyloid was absent from liver, paralleling human disease.

**Fig. 3:**
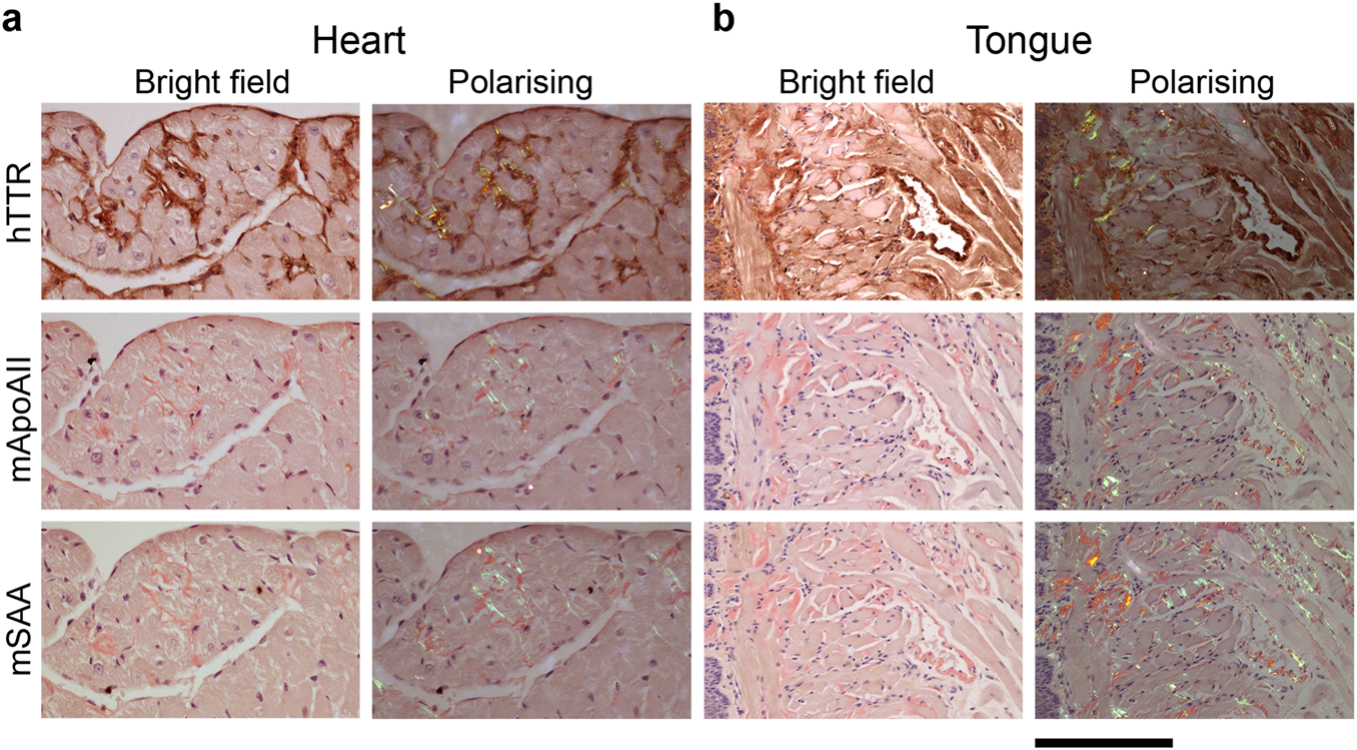
Immunohistochemical demonstration of ATTR amyloid in the transgenic model. To determine the amyloid type, heart (**a**) and tongue (**b**) were stained with anti-human TTR, anti-mouse apoAII or anti-mouse SAA antisera, as indicated, followed by alcoholic alkaline Congo red. Bright field images show the immunohistochemical signal (brown), and faint red Congo red stained amyloid in a representative amyloidotic line N4 mouse (*n* = 15). The amyloid deposits exhibited the characteristic red-green birefringence when viewed by polarising microscopy. The coincidence of TTR signal and amyloid, together with the absence of apoAII and SAA signal, demonstrates that the amyloid is of ATTR type. In places, the intense anti-TTR staining obscured the Congo red staining, the extent of which is clearly visible in the closely adjacent sections stained for apoAII and SAA. Scale bar, 100 µm (**a**) and 200 µm (**b**). hTTR human TTR, mApoAII mouse apolipoprotein A2, mSAA mouse serum amyloid A protein.

In the ATTR amyloid-injected *TTR*^S52P^ transgenic mice, the distribution of amyloid was clearly distinct from those observed in murine AA and AApoAII amyloidosis, the types of amyloidosis that occur naturally, both of which feature prominent splenic amyloid as well as hepatic amyloid. Consistent with this, immunohistochemical staining of adjacent sections of the amyloidotic tissues from 19 line O5 and 15 line N4 seeded transgenic mice, using antibodies to human TTR, mouse SAA and mouse apoAII, confirmed that the amyloid deposits were composed of human TTR in every case (e.g. Fig. 3).

Proteomic mass spectrometry analysis of amyloid deposits obtained by laser capture microdissection of tissue sections, further confirmed the amyloid fibril type as TTR^S52P^ in each of the six amyloidotic animals analysed (Table 1). Neither mouse apolipoprotein AII nor serum amyloid A protein was identified in any sample, further ruling out AApoAII and AA amyloidosis. Proteins comprising the typical ‘amyloid signature’ detected in proteomic analysis, and used to aid diagnosis of amyloidosis^34–36^, were present in each of the amyloidotic animals, along with other proteins that are also known to be amyloid-associated (Table 1). These findings independently confirm the presence of ATTR amyloid in the mice analysed.

**Table 1:** Proteomic analysis of amyloid deposits in human *TTR*^S52P^ transgenic mice.

	Mouse (line)	Tissue	Human TTR^S52P^		Amyloid-signature proteins Mascot score (Matches/USP)			
			Mascot score (Matches/USP)	Coverage (%)	ApoA4	ApoE	SAP	Vitronectin
**Seeded, amyloidotic**	1.14 (O5)	Tongue	341 (10/7)	85	27 (2/2)	170 (6/6)	–	103 (5/4)
	2.10 (O5)	Tongue	459 (13/8)	75	33 (1/1)	91 (3/3)	–	63 (3/3)
	14.4 (N4)	Heart	1793 (60/8)	79	148 (7/5)	22 (1/1)	39 (1/1)	54 (3/2)
	14.4 (N4)	Tongue	1431 (56/8)	79	170 (6/5)	26 (1/1)	–	53 (2/2)
	12.9 (N4)	Tongue	6354 (185/15)	100	782 (24/16)	571 (23/12)	201 (6/5)	250 (11/7)
	12.15 (N4)	Tongue	6324 (184/14)	96	904 (28/17)	699 (28/12)	236 (8/6)	404 (15/6)
	12.22 (N4)	Tongue	4572 (132/14)	95	647 (24/16)	465 (20/11)	232 (8/6)	224 (10/5)
**Unseeded non-amyloidotic controls**	164894 (N4)	Tongue	81 (3/2)	20	–	–	–	–
	22.13 (N4)	Tongue	40 (1/1)	10	–	–	–	–
	22.15 (N4)	Tongue	32 (2/2)	28	–	–	–	–

LC-MS/MS analysis of laser capture microdissected amyloid (seeded, amyloidotic) or equivalent control samples (unseeded, non-amyloidotic). Mascot analysis identifies human TTR in the microdissected material; the 52P-containing peptides 49–70 and 49–76 were present in most samples. The Mascot scores are consistent with the relative amounts of amyloid in the tissues (small in seeded line O5 mice, large in seeded line N4 mice and none in unseeded line N4 mice). Our normal minimum acceptance score for clinical amyloid proteins^96^ is 80, and for signature proteins it is 20. Human TTR was detected in the control samples, probably reflecting the high concentration of TTR in the circulation of line N4 mice, but the scores were very low. Identification of multiple amyloid-associated proteins in the seeded, but not unseeded control mouse samples is consistent with the amyloid content of the deposits.

*USP* unique significant peptide, *ApoA4* apolipoprotein A4, *ApoE* apolipoprotein E, *SAP* serum amyloid P-component.

### Specificity of seeding

Seeding with ATTR amyloid material extracted from transgenic mouse tissues was also able to initiate ATTR amyloid deposition in otherwise untreated *TTR*^S52P^ transgenic animals. Fifteen line N4 transgenic mice were injected with extracts of heart or tongue from an amyloidotic line N4 mouse, in which amyloid deposition had been triggered by administration of patient-derived ATTR^S52P^ amyloid material. Four months later, every mouse that had received extracts of ATTR amyloid-containing mouse heart (7/7) or tongue (8/8) had abundant amyloid deposits in both heart and tongue, but not in spleen or liver. In contrast, there was no ATTR amyloid deposition in any site in any line N4 mouse four months after administration of extracts of mouse AA amyloid (0/8), demonstrating that seeding was amyloid type specific.

### Effects of *ttr* genotype and sex on amyloid deposition

The 39 line O5 mice which developed human TTR amyloid included mouse *ttr* wild-type animals as well as mice heterozygous or homozygous for the mouse *ttr* gene knockout (supplementary table 1), so mouse TTR evidently had no discernible effect on amyloid deposition. A sex difference in amyloid deposition was apparent in line O5 mice, with amyloid present in 34 out of 37 males but only 5 out of 14 females (supplementary table 1). In contrast, 2 months after seeding of line N4 mice, which express >10-fold more human TTR than line O5, amyloid was found in hearts and tongues of all mice of both sexes (*n* = 10 each sex), though the amount of amyloid deposited in males was greater than in females. In both lines O5 and N4, the concentration of human TTR was lower in females than in males, by 50% and 30%, respectively (*P* < 0.0001 for both lines, two-tailed Mann–Whitney test; Fig. 1), so these findings are consistent with the well-established relationship between the amyloid precursor protein concentration and efficiency of amyloid deposition, though they do not rule out an effect of gender operating through another mechanism. To separate possible effects of sex from effects of TTR concentration, we directly compared amyloid deposition in line N1 females and line O5 males. The concentrations of human TTR in line N1 females were greater than in line O5 males (*P* = 0.0005, two-tailed Mann–Whitney test; Fig. 1). Nevertheless, when analysed 4 months after seeding, a greater proportion of line O5 males contained amyloid than females of line N1 (6 of 8 line O5 male mice vs 1 of 8 female mice; supplementary table 2, *P* = 0.041, Fisher’s exact test), indicating that there may indeed be an effect of gender that is independent of the human TTR concentration.

### TTR cleavage and amyloid deposition

**Fig. 4:**
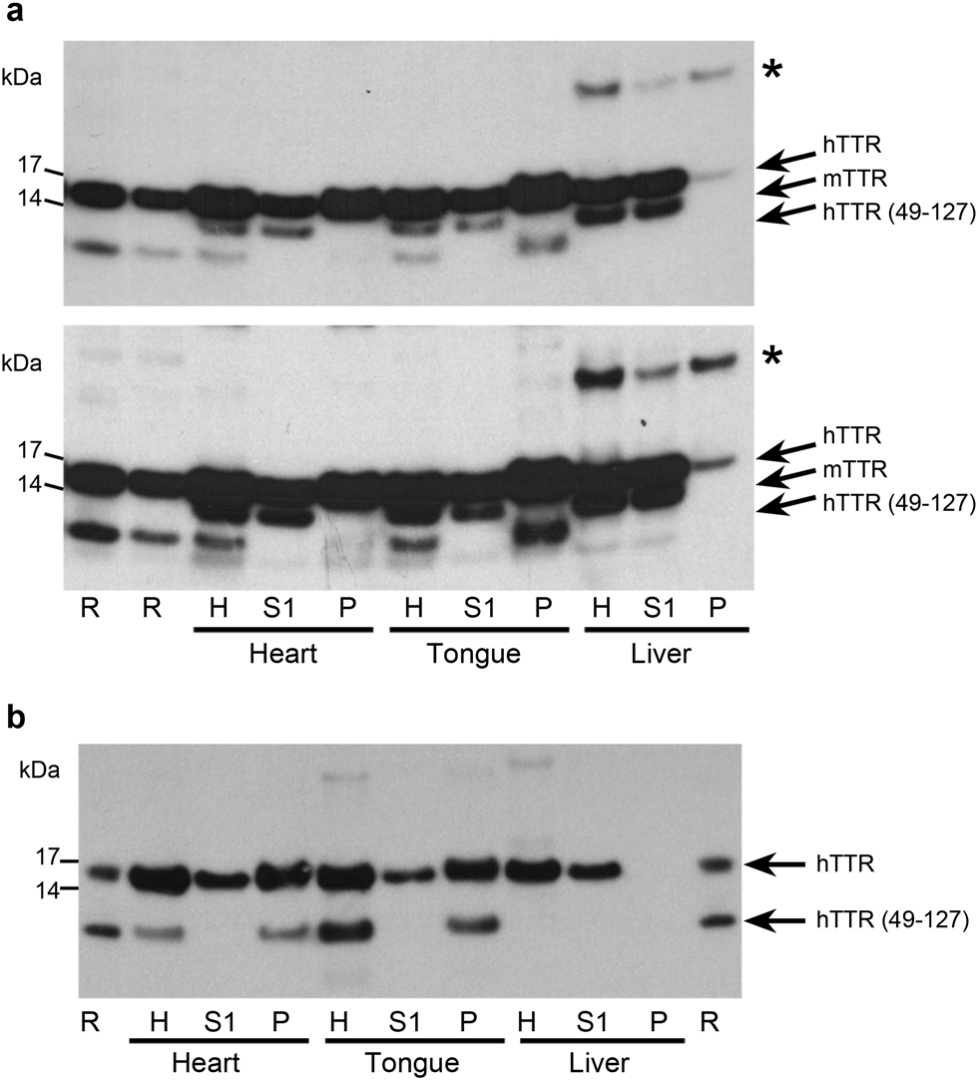
Presence of cleaved human TTR in transgenic human ATTR amyloid. Homogenates of heart, tongue and liver from amyloidotic line O5 and line N4 mice (**a** and **b**, respectively) were fractionated into soluble and insoluble fractions, and analysed by western blotting. Lanes labelled H contain unfractionated homogenates, lanes labelled S1 contained the soluble fraction (first supernatant), and lanes labelled P contained the insoluble fraction (pellet). The line O5 mouse (**a**) was homozygous wild-type for mouse *ttr* and the line N4 mouse (**b**) was homozygous for the mouse *ttr* knockout allele. The same proportion of the entire sample was loaded in each lane; two exposures are shown in **a** to reveal all relevant bands. Data are representative of *n* = 5 (**a**) and *n* = 2 (**b**) biological replicates. Lanes labelled R contained recombinant human TTR^S52P^ that had undergone limited tryptic cleavage; the positions of full-length TTR protomer, the 49–127 cleavage product and mouse TTR are indicated. Glycosylated human TTR protomer is indicated by asterisks. Source data are provided as a Source Data file.

In most cases of human ATTR amyloidosis, the amyloid fibrils contain a mixture of intact TTR protomers and a C-terminal proteolytic cleavage fragment comprising TTR residues 49–127 (ref. ^37^). This observation led to the discovery that cleavage of native TTR at the 48–49 peptide bond, dramatically destabilises the tetrameric assembly leading, under agitation, to the formation of abundant authentic TTR amyloid fibrils in vitro^25,26^. The predominant form of human TTR detected in western blot analysis of homogenates of tissue from the seeded amyloidotic transgenic mice was the full-length TTR protomer (Fig. 4). However, in homogenates of amyloidotic heart and tongue, the 49–127 fragment was always present as a minor component. To separate soluble and insoluble material (containing native TTR and TTR amyloid, respectively), the homogenates were fractionated by centrifugation. While full-length TTR protomers were present in both fractions, the residue 49–127 fragment was exclusively present in the insoluble fractions of the homogenates of amyloidotic heart and tongue, demonstrating that it is an integral component of the amyloid fibrils themselves. These findings are consistent with the pivotal importance of the specific cleavage at the 48–49 peptide bond in the pathogenesis of TTR amyloid deposition. The 49–127 fragment was not detected in livers from amyloidotic mice, nor in heart, tongue or liver of old transgenic mice that had not been seeded, all of which lacked detectable amyloid (Supplementary Fig. 8). Mouse TTR was detected in supernatants of amyloid-containing heart and tongue homogenates, and was thus soluble, but was absent from the insoluble fractions, showing that it was not present in the amyloid fibrils.

**Fig. 5:**
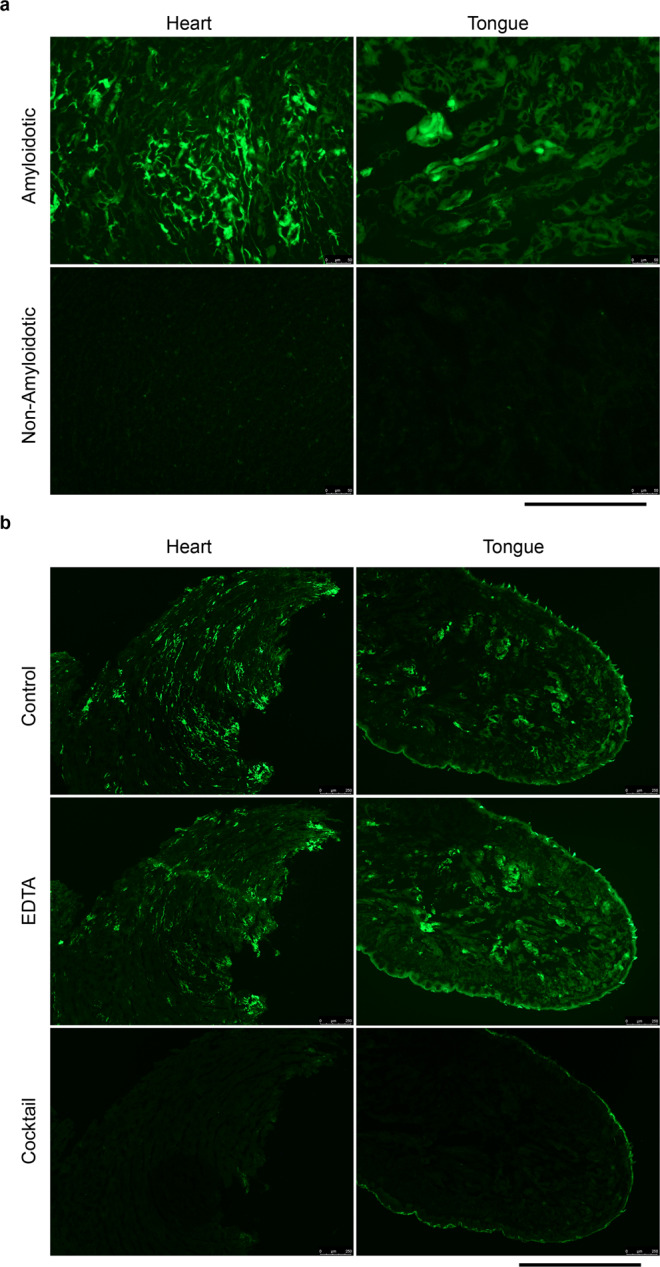
Amyloid-associated protease activity. Protease activity in heart and tongue was visualised by in situ zymography using DQ-gelatin substrate overlaid on unfixed cryosections. **a** protease activity was readily detected in hearts and tongues of amyloidotic mice, but not of control mice. **b** the amyloid-associated protease activity was insensitive to inhibition by EDTA, but was completely inhibited by a cocktail of serine protease inhibitors (aprotinin, PMSF and Pefabloc SC). The protease activity was associated with amyloid deposits, but the activity was not as extensively distributed as the amyloid (Fig. 2c). Scale bars: 250 µm (**a**); 1 mm (**b**). Data are representative of a minimum of two technical replicates of two biological replicates.

**Fig. 6:**
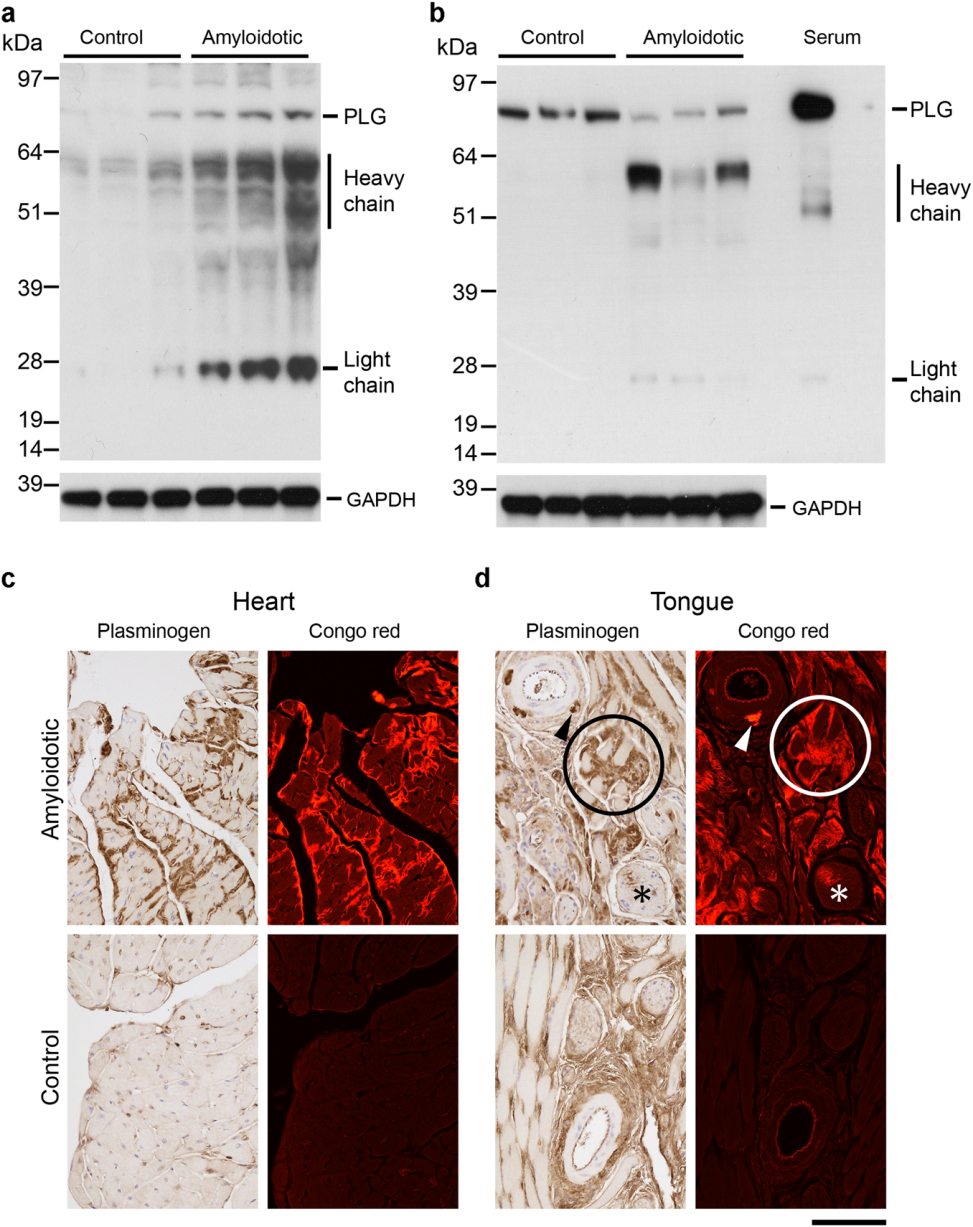
Association of plasmin(ogen) with amyloid deposits. **a**, **b** Western blots probed with an affinity-purified anti-plasminogen (PLG) antiserum, comparing amyloidotic and control heart (**a**) and tongue (**b**) from three different mice. The amounts of plasmin heavy and light chains in amyloidotic hearts and tongues was greater than in the control tissues. GAPDH was used as a sample processing control for the tissue extracts. **c**, **d** Adjacent tissue sections of heart (**c**) or tongue (**d**) of amyloidotic and control non-amyloidotic mice were probed with an affinity-purified anti-plasminogen antiserum, or stained with Congo red. **c** In amyloidotic heart, the pattern of immunoreactivity closely parallels the distribution of amyloid deposits. In control heart, the signal is much lower and almost exclusively within the vasculature. **d** In control tongue, moderate immunoreactivity is evident in connective tissue, consistent with the presence of plasminogen in the extracellular space. In amyloid-containing tongue, additional strong immunoreactivity was evident in the same distribution as the amyloid seen here, for example, surrounding muscle fibres (circled), in the adventitia of an arteriole (arrowhead), and within a small nerve (asterisk). Images in **c** and **d** are representative of findings in three different mice. Scale bar: 100 µm. Source data are provided as a Source Data file.

To investigate the relationship between amyloid deposits and proteolytic activity, we performed in situ zymography assays on hearts and tongues of amyloidotic and control mice, which revealed much greater protease activity in amyloidotic tissues (Fig. 5a). The protease activity was less extensively distributed than the amyloid, but it precisely co-localised with amyloid in both hearts and tongues, showing that amyloid was a site of active proteolysis. The protease activity was insensitive to EDTA, but the zymography signal was quantitatively inhibited by a cocktail of aprotinin, pefabloc and PMSF (Fig. 5b). This indicates that the protease(s) responsible for the amyloid-associated activity was a serine protease, and rules out the possibility that the cryptic metalloprotease activity of TTR (refs. ^38,39^) was responsible for the signal. On the basis of bioinformatic and in vitro studies, the serine protease plasmin has been identified as a candidate physiological protease for TTR cleavage leading to amyloid deposition^27,40^. Active plasmin comprises two polypeptides (heavy and light chains) generated by cleavage of the inactive proenzyme, plasminogen. Western blot analysis (Fig. 6a, b) showed the presence of plasmin heavy and light chains in hearts and tongues of amyloidotic mice, while in control mouse hearts and tongues, they were not detected, or were much less abundant than in amyloidotic mice. Immunohistochemistry for plasmin/plasminogen in amyloidotic tissues gave very strong signal that co-localised with amyloid in heart and tongue (Fig. 6c, d). Together, these data show that plasmin was enriched in amyloid deposits.

**Fig. 7:**
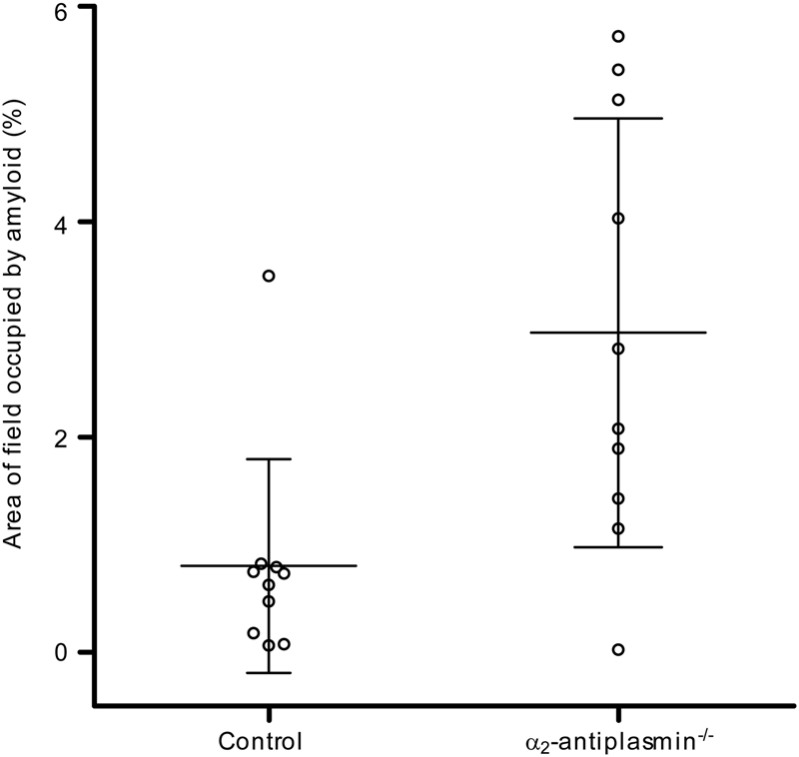
Effect of α_2_-antiplasmin deficiency on amyloid deposition. Amyloid deposition was enhanced by α_2_-antiplasmin deficiency (*P* = 0.0068, Mann–Whitney test, two-tailed). Estimates of lingual amyloid content (individual values, mean and SD) are shown for male line N4 human *TTR*^S52P^ mice that were wild-type (control) or homozygous for an α_2_-antiplasmin knockout allele (*n* = 10 each group). The tissues were collected 2 months after amyloid deposition was seeded by injection of human TTR^S52P^ amyloidotic spleen homogenate. Similar results were obtained when comparing α_2_-antiplasmin deficient (homozygous knockout) with control heterozygous α_2_-antiplasmin knockout male mice (*P* = 0.0047, Mann–Whitney test, two-tailed, *n* = 7 controls, 6 knockouts). Source data are provided as a Source Data file.

To test directly whether plasmin activity contributes to the formation of ATTR amyloid in vivo, we evaluated the effect of knockout of the specific plasmin inhibitor α_2_-antiplasmin (Serpinf2) on ATTR amyloid deposition. Greater amyloid deposition was evident in line N4 human TTR^S52P^ transgenic mice that were also homozygous for a knockout of α_2_-antiplasmin^41^ than in control line N4 TTR^S52P^ transgenic mice that were heterozygous for the α_2_-antiplasmin knockout (*P* = 0.0047, two-tailed Mann–Whitney test) or homozygous α_2_-antiplasmin wild-type (*P* = 0.0068, two-tailed Mann–Whitney test) (Fig. 7). Inhibition of plasmin activity is the only significant physiological activity of α_2_-antiplasmin (ref. ^42^), so the finding that α_2_-antiplasmin knockout increased ATTR amyloid deposition demonstrates that plasmin activity promotes ATTR amyloid deposition in vivo.

**Fig. 8:**
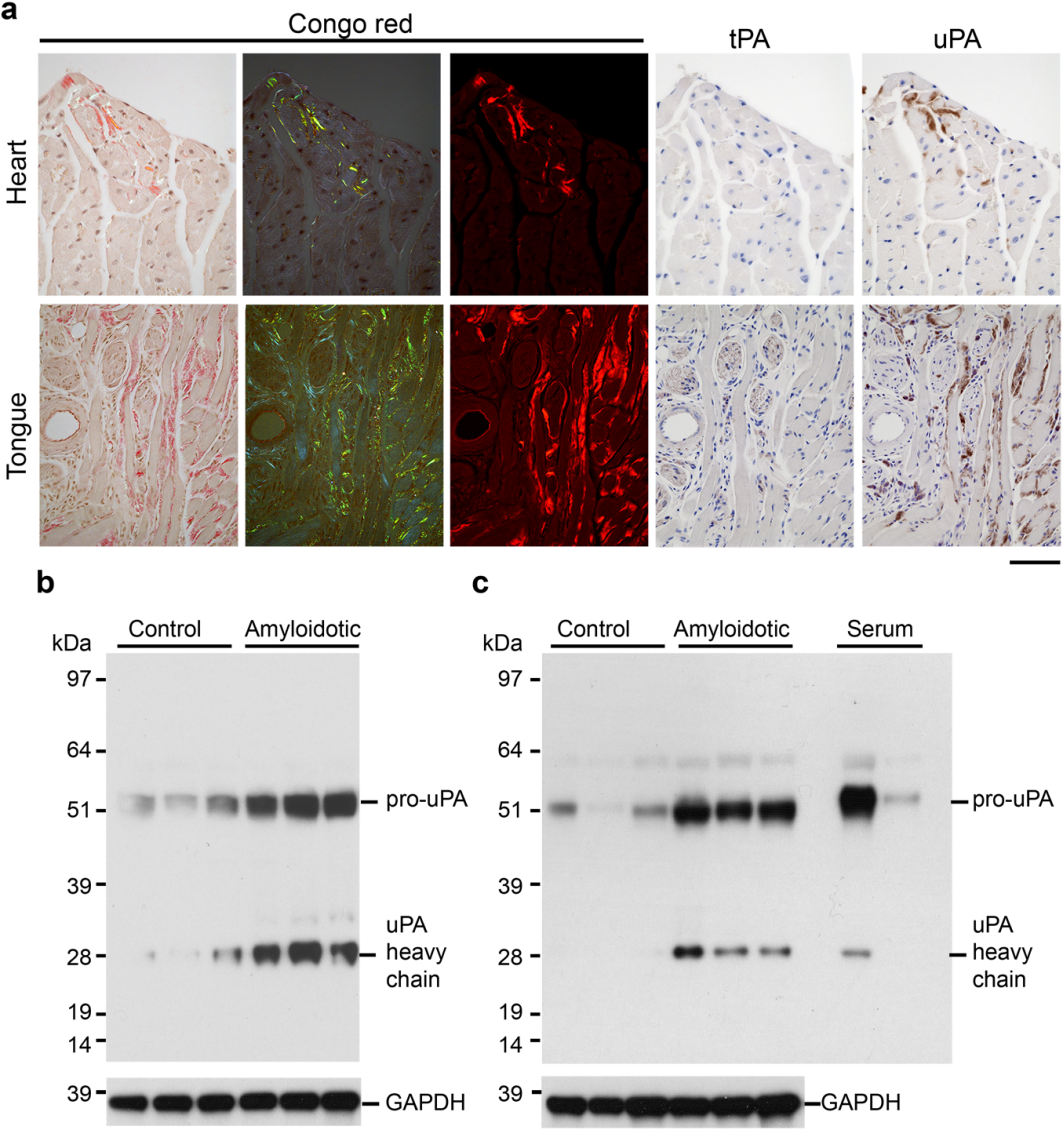
Relationship between plasminogen activators and amyloid. **a** Adjacent sections of human TTR^S52P^ amyloid-containing heart and tongue stained with Congo red to show amyloid or probed with anti-tPA or anti-uPA affinity-purified antisera (representative of results seen with six mice). The patterns of anti-tPA immunoreactivity were indistinguishable from those of normal control tissues (Supplementary Fig. 9). The amyloid deposits in heart and tongue were strongly immunoreactive when probed for uPA. Additional strong immunoreactivity in tongue was observed in mast cells, indistinguishable from that seen in normal control tissue (Supplementary Fig. 9). **b**, **c** Western blot showing greater amounts of uPA in amyloidotic hearts (**b**) and tongues (**c**) than in controls (*n* = 3 amyloidotic and 3 control mice). Amyloidotic tissues contained more pro-uPA, the precursor of active uPA, as well as the heavy chain of active uPA; the antibody used for this analysis does not react with uPA light chain. GAPDH was used as a sample processing control for the tissue extracts. Scale bar: 50 µm (heart); 100 µm (tongue). Source data are provided as a Source Data file.

There are two main physiological activators of plasminogen: tissue-type plasminogen activator (tPA) and urokinase-type plasminogen activator (uPA) (Rijken and Lijnen^43^). Tranexamic acid (TXA) is a drug which suppresses fibrinolysis by inhibiting tPA-mediated plasminogen activation, and which is commonly used e.g. during surgery and for treatment of menorrhagia^44^. To test the potential of TXA to inhibit amyloid deposition, groups of 10 male line N4 transgenic mice were seeded and treated with TXA in their drinking water continuously for 2 months starting 2 days before seeding, or one or two months after seeding, and compared with contemporaneous groups of seeded mice maintained on water. None of these treatments with TXA had any detectable effect on the extent of amyloid deposition (*P* = 0.123; *P* = 0.6787; *P* = 0.3097, respectively; two-tailed Mann–Whitney tests), showing that tPA-mediated plasminogen activation is not a major driver of amyloid deposition in the transgenic model. Consistent with this, no difference was observed in the amount and pattern of immunohistochemical staining for tPA in amyloidotic and control tissues (Fig. 8a and Supplementary Fig. 9). In contrast, much greater uPA staining was observed in amyloid-containing heart and tongue (Fig. 8a) compared with control tissues (Supplementary Fig. 9), with strong staining specifically co-localised with amyloid (Fig. 8a). Western blot analysis showed that amyloidotic hearts and tongues contained more uPA than control tissues and contained cleaved uPA, indicating that the uPA had been activated (Fig. 8b, c).

## Discussion

There has long been a need for a robust, reproducible and tractable experimental model of human ATTR amyloidosis for investigation of pathogenetic mechanisms as well as therapeutic approaches and diagnostic methods, and this need remains despite many attempts in a variety of systems^45^. Previous human *TTR* transgenic mouse models have succeeded in expressing the protein but have been associated with only minor ATTR amyloid formation, even after attempted seeding^46–54^. Although more substantial amyloid deposits have been reported, these have all occurred in aged mice, and have not rigorously been confirmed to be of ATTR rather than apoAII or AA types, both of which may develop ‘spontaneously’ in ageing experimental mice^50,51,55–59^. In marked contrast, the mice reported here consistently accumulate genuine, Congophilic, human TTR^S52P^ amyloid fibrils, predominantly in the heart and tongue. While lingual amyloid is usually not observed or reported in ATTR amyloidosis, it is a well-documented feature of the disease in some patients^3,60–63^. The model recapitulates key features of the human disease: its delayed onset, the presence of the TTR residue 49–127 proteolytic fragment in the amyloid fibrils, the predilection for amyloid deposition in sites of continuous or frequently repeated mechanical activity, and the greater prevalence of cardiac ATTR amyloidosis in males.

In all forms of amyloidosis, the kinetics of amyloid deposition depend on the supply of the respective amyloid precursor protein, and very high-level expression of TTR in line N4 mice here was associated with the most abundant amyloid deposition. There was a striking non-linear relationship between circulating TTR concentrations in the mice and the amounts of ATTR amyloid deposited. The difference in amyloid loads between comparably treated mice of lines N4 and O5 far exceeded the 10- to 20-fold difference in TTR concentrations between them, by at least an order of magnitude. This suggests that modest TTR concentration differences may have disproportionate effects on the rates of net amyloid deposition. The ~20% higher circulating TTR concentrations in men than in women^64^ may therefore be an important factor in the higher incidence of cardiac ATTR amyloidosis in men, but other findings in the mouse model suggest that there may be additional gender effects that are independent of TTR concentration, the nature of which are as yet unknown.

Human ATTR amyloidosis is almost never diagnosed before the third decade, even in individuals with the most ‘aggressive’ TTR gene mutations, including *TTR*^S52P^. *TTR*^V122I^ and *TTR*^V30M^, the most prevalent amyloidogenic *TTR* mutations globally, are incompletely penetrant in some families and may not cause disease even in individuals homozygous for the pathogenic allele, while wild-type ATTR amyloidosis is exclusively a disease of late middle age and beyond. Very long term, abundant expression of amyloidogenic TTR alone is thus not necessarily sufficient to initiate TTR amyloid fibrillogenesis in vivo in humans. Similarly, even lifelong very abundant expression of the highly amyloidogenic human TTR^S52P^ variant did not produce amyloid in the transgenic mice. The efficacy of isolated amyloid fibrils as seeds for in vivo amyloidogenesis was first demonstrated in 1984 in murine systemic AA amyloidosis^65^ and has been demonstrated in vitro for all known amyloid fibril forming proteins^27,66–69^. The efficacy of ex vivo human TTR^S52P^ fibrils in initiating amyloid deposition in the present transgenic mice therefore strongly suggests that a stochastic seeding event, whether internally or externally derived, plays a key role in the human disease. The events that initiate amyloid deposition in the human disease are unknown and are a particularly intractable problem, though relevant information is likely to emerge in future, e.g. from genetic and epidemiological studies of susceptibility to ATTR amyloidosis. Our new model has potential for validation of such findings and, because spontaneous amyloid deposition does not occur in the mice, may enable factors that promote the initiation of ATTR amyloid deposition to be distinguished from those that modify disease progression alone.

Even after seeding with authentic ex vivo human TTR^S52P^ amyloid fibrils, the appearance of amyloid deposition in the mice was delayed and progression was slow, just as in clinical ATTR amyloidosis. Although we found that mice of lines O5 and N1 did reproducibly accumulate amyloid, the deposits were very small and sparsely distributed, even many months after seeding. In line N4, by contrast, the first amyloid deposits appeared within 4 weeks, and from 2 months were readily detectable, particularly in males, and clinically significant amounts accumulated over subsequent months. For many uses of the model, a fortuitous but important advantage of the lack of any spontaneous amyloid deposition is that injection of exogenous ATTR amyloid seeds synchronises the commencement of amyloid deposition, eliminating variability that would result from stochastic endogenous seeding. The finding that ATTR amyloid-containing transgenic mouse tissue can be used to seed deposition removes the requirement for seeding with very limited availability of patient-derived amyloid.

The S52P substitution in human TTR increases the susceptibility of the protein to tryptic cleavage of the 48–49 peptide bond, generating the residue 49–127 fragment that is typically a prominent component of human cardiac ATTR amyloid deposits^25,37^. In contrast with the intact protein, partially cleaved TTR^S52P^ readily forms amyloid in vitro^25^. Other amyloidogenic variants tested, as well as wild-type human TTR, are not susceptible to this limited proteolysis unless they are also subjected to shear stress^25,26^. Once cleaved in these mechano-enzymatic conditions, they also readily form amyloid fibrils in vitro at physiological pH and salt concentration^26^, yielding fibrils with very similar morphology and biophysical properties to natural ex vivo human ATTR amyloid fibrils^26,40^. Clinical wild-type ATTR amyloid presents almost exclusively with cardiac amyloidosis, which is often presaged by carpal tunnel syndrome due to deposits in the flexor retinaculum and/or flexor tenosynovium^70^, and is usually accompanied by amyloid in skeletal muscle^14^. The accumulation of amyloid in these sites of mechanical stress is consistent with a mechano-enzymatic mechanism for amyloid formation. Several findings presented here support this hypothesis and identify plasmin as a physiological protease that contributes to ATTR amyloid deposition: the presence of abundant serine protease activity within amyloid deposits, increased ATTR amyloid deposition resulting from α_2_-antiplasmin deficiency, the presence of immunoreactive plasminogen/plasmin in amyloid deposits, and the accumulation of plasmin heavy and light chains in amyloid-containing hearts and tongues.

Most proteases, including plasmin and uPA (but not tPA) are synthesised as inactive zymogens which require specific activation, and protease activity is normally rapidly terminated by specific protease inhibitors, augmented by abundant general protease inhibitors. Active plasmin is generated by cleavage of plasminogen, normally by tPA or uPA. In fibrinolysis, tPA and plasminogen both bind to fibrin clots bringing them into close proximity, thereby promoting cleavage of plasminogen by tPA, to yield active plasmin. Tranexamic acid, a widely used medicine, inhibits plasmin activation by tPA by inhibiting the binding to fibrin. The lack of a detectable effect of tranexamic acid on amyloid deposition in the mouse model indicates that this pathway of plasmin activation did not contribute appreciably to amyloid formation. In contrast, the accumulation of cleaved uPA in amyloid is consistent with local uPA-mediated generation of plasmin at the sites of amyloid deposition. Plasminogen activation by uPA is known to be required for normal tissue remodelling, including in myocardial infarct healing^71,72^, skeletal muscle regeneration^73,74^ and vascular wound healing^75–77^. Furthermore, uPA and plasmin have also been shown to mediate the development of cardiac hypertrophy and fibrosis induced by experimental treatments such as transverse aorta constriction (widely used as a model for aortic stenosis), hypoxia or pharmacologically induced hypertension^78–82^. Thus, uPA-mediated plasminogen activation is known to have normal physiological functions and pathogenetic roles at sites of ATTR amyloid deposition. This is consistent with TTR being cleaved (and deposited in amyloid) as a result of spill-over of normal physiological uPA/plasmin activity. Both uPA and vitronectin, a prominent component of all amyloid deposits, bind with high affinity to urokinase receptor (uPAR), forming a ternary complex^83,84^. uPAR exists as a GPI-linked cell surface molecule and may be shed from the cell surface, as so-called soluble uPAR (suPAR), which has been proposed as a prognostic biomarker for a wide range of conditions^85^. This raises the intriguing possibility that, once the first amyloid deposits appear, these interactions specifically concentrate uPA in amyloid and create a local proteolytic environment within ATTR amyloid deposits which accelerates the further conversion of circulating TTR into amyloid.

## Methods

### Transgenic mice

Following site-directed mutagenesis^86^ to create the S52P mutation, the transgene construct was built by Gibson assembly^87^ of an albumin enhancer/promoter cassette^28^ with the human TTR^S52P^ gene. Following sequence verification, the transgene fragment was isolated from the plasmid vector and microinjected into pronuclei of 1-cell eggs. Transgenic mice were identified by PCR with primers ACCCAAGGCTTTTGCCTAAT and ATTGCTTCCCATTTGACTGC. Generation of the mouse *ttr* and *Serpinf2* (α_2_-antiplasmin) knockout mice were described previously^41,88^. The mice were maintained on the C57BL/6J background, and all *TTR*^S52P^ transgenic mice were hemizygous for the transgene. Line N4 on the α_2_-antiplasmin knockout background has been deposited in the European Mutant Mouse Archive with accession code EM:13232.

In each line, the human *TTR*^S52P^ transgenic mice were consistently smaller than non-transgenic control mice (Supplementary Fig. 10) but the reasons for this are not known. TTR binds and transports thyroid hormones but, although both total T4 and free T4 were increased in the transgenic mice (Supplementary Fig. 11a, b), TSH concentrations were normal (Supplementary Fig. 11c). The growth reduction is therefore probably not attributable to disturbance of thyroid function. The avid specific binding of retinol binding protein (RBP) and TTR in the plasma retains the low molecular weight RBP, and thus its transported retinol, in the circulation. The concentrations of RBP and retinol are very low in *ttr*^−/−^ mice (Supplementary Fig. 11d and Episkopou et al.^88^) but expression of human TTR^S52P^ partially restored RBP concentration towards normal (Supplementary Fig. 11d), so the reduced growth is probably not due to retinol deficiency.

For seeding of amyloid deposition, extracts of TTR amyloid-containing spleen from an ATTR^S52P^ patient or heart or tongue from an ATTR^S52P^ amyloidotic mouse were prepared by homogenisation in 50 volumes sterile phosphate buffered saline and slow speed centrifugation (1000 × *g*; 10 mins). to pellet any remaining tissue fragments. Limited amounts of human ATTR amyloid-containing tissue suitable for use to seed amyloid deposition are available on request.

Mice were maintained at 20 °C to 24 °C, 45 to 65% relative humidity and a 12:12 h light:dark cycle. All animal studies were ethically reviewed and approved by the UCL Royal Free Campus Animal Welfare and Ethical Review Body and carried out in accordance with the provisions of the United Kingdom Animals (Scientific Procedures) Act 1986. The amyloid-containing human spleen tissue was donated with informed written consent (in accordance with the Helsinki Declaration), and was used in accordance with approval granted by the Ethics Committee of the Royal Free Hampstead NHS Trust.

### Biochemical assays

Human TTR in mouse sera was quantified by electroimmunoassay in 1% w/v agarose gels prepared in barbitone–EDTA buffer pH 8.6 using monospecific rabbit anti-human TTR antiserum (Dako A0002), calibrated with pure wild-type human TTR. We found that quantification of human TTR in the serum of transgenic mice by electroimmunoassay was sensitive to the presence of mouse TTR protein, resulting in overestimation of the human TTR concentration. Comparison of western blot assay and analysis of sera from line O5 human *TTR* transgenic mice on the wild-type, hemizygous and homozygous mouse *ttr* knockout backgrounds, demonstrated that the overestimation was due to the known formation of mixed mouse-human TTR tetramers^29,30^. Mouse TTR evidently interferes in electroimmunoassay of human TTR, which is dependent on electrophoretic mobility and immunoprecipitation of the native protein^89^, but does not interfere in western blotting where these variables are eliminated (Supplementary Fig. 12a, b).

Tissue was homogenised using a TissueLyser II (Qiagen) in 10 volumes (w:v) ice-cold homogenization buffer (10 mM Tris-Cl; 10 mM EDTA; 140 mM NaCl; 0.1% NaN_3_ pH8.0, with addition of Roche Complete Protease Inhibitor Cocktail to 1x and PMSF to 1.5 mM immediately prior to use). Fractionation of soluble and insoluble material was performed by 4 sequential centrifugation steps (13,500 × *g*; 30 mins; 4 °C); at each step, after removal of the supernatant and addition of the starting volume of homogenization buffer, pellets were re-homogenised. Under these conditions, the soluble TTR was quantitatively removed after the second round of centrifugation.

Proteins were electrophoresed on NuPAGE Novex 12% or 4–12% Bis-Tris gels alongside a pre-stained protein standard (SeeBlue Plus2, Life Technologies, UK) using MES-SDS buffer (50 mM MES, 50 mM Tris base, 0.1% SDS, 1 mM EDTA, pH 7.3). After SDS-PAGE electrophoresis, the gel was transferred to polyvinylidene difluoride (PVDF) membranes. Blots were probed for TTR with 3.1 μg/ml sheep anti-human TTR (The Binding Site, Birmingham, UK), using HRP-labelled rabbit anti-sheep (1:2000; Dako P0163) secondary antibody. Plasminogen and plasmin were probed for using 0.42 μg/ml rabbit anti-mouse plasminogen (Molecular Innovations ASMPLG-GF-HT) and HRP-labelled Goat Anti-Rabbit secondary antibody (1:2000; Dako P00048). Urokinase/uPA was probed using 0.8 μg/ml mouse anti-uPA (Santa Cruz sc-59727) and Clean-Blot™ IP Detection Reagent (HRP) (Thermo-Fisher 21230) as secondary. GAPDH was probed using rabbit monoclonal anti-GAPDH (1:1000; Cell Signaling Technology), and HRP-labelled Goat Anti-Rabbit secondary antibody (1:2000; Dako P00048). ECL reagent (RPN2109) and Hyperfilm ECL (28-9068-36) were used for detection.

De-glycosylation was performed with PNGaseF (New England Biolabs) essentially as recommended by the manufacturer: 0.4 µl of serum (~20 µg of total protein) was denatured in 10 µl glycoprotein denaturating buffer (0.5% SDS, 40 mM DTT) at 100 °C for 10 min. After addition of GlycoBuffer 2 and NP-40 and 500 units PNGaseF, reactions were incubated for 1 h or 24 h at 37 °C. Control reactions were identical with the omission of the enzyme.

Assays of total T4, free T4, TSH, and retinol binding protein 4 were performed using commercial ELISA kits (Calbiotech T4044T-96T and F4223T, Cloud-Clone Corp. CEA463Mu, and Bio-Techne MRBP40, respectively) according to the manufacturers’ instructions.

### Histology

Formalin-fixed wax-embedded tissues were sectioned at 6 µm, stained with alkaline alcoholic Congo red^90^ and viewed under strong cross polarised light, or by epifluorescence microscopy (excitation: 497 nm; emission: 614 nm). For amyloid typing by immunoperoxidase staining, 3 µm wax sections were pre-treated sequentially with 1% sodium metaperiodate, 0.1% sodium borohydride and 6 M guanidine hydrochloride, for antigen retrieval. After blocking endogenous peroxidase with 0.6% H_2_O_2_, sections were incubated with 2.5% normal horse serum (Vector labs.), and then with primary antibody in Dako antibody diluent for 1 h at room temperature or at 4 °C overnight. Primary antibodies were from Dako (rabbit anti-TTR; diluted 1:4000), R&D systems (goat anti-SAA; diluted 1:100) and from Prof. Keiichi Higuchi (rabbit anti-ApoAII; diluted 1:4000). Peroxidase-conjugated secondary antibodies (ImmPRESS, Vector labs) were incubated with sections for 30 min., detected using Metal-Enhanced DAB Substrate and Stable Peroxide Substrate Buffer (Pierce) and counterstained with Mayer’s haematoxylin. The specificity of staining was confirmed by the use of positive control sections of confirmed ATTR amyloid, as well as sections from mice with AA amyloidosis, AApoAII amyloidosis and non-amyloidotic mouse tissue. Laser capture microdissection and proteomic mass spectrometry analysis of Congo red-stained amyloid in tissue sections was performed essentially as described^91^. Immunoperoxidase staining for plasmin(ogen), tPA and uPA was performed essentially as above but with antigen retrieval by incubation in basic (plasminogen, uPA) or acidic (tPA) antigen retrieval buffers (R&D systems). The antigen affinity-purified polyclonal anti-plasminogen, tPA and uPA antibodies were from Molecular Innovations Inc., and were used at 0.15 µg/ml (plasminogen), 5 µg/ml (tPA) and 1.5 µg/ml (uPA). These antibodies showed the expected pattern of staining in control tissues (vascular and extracellular space (plasminogen); endothelial cells, nerves and mast cells (tPA); renal tubules and mast cells (uPA)). Further, the anti-plasminogen antibody showed the expected pattern on western blots of serum and tissue extracts. In situ zymography was performed^92^ on 10 µm cryosections of unfixed tissue. Sections were thawed, overlaid with 100 µg/ml DQ-gelatin (ThermoFisher), 1% low gelling temperature agarose in PBS and incubated overnight at room temperature. For inhibitor studies, the sections were in addition, pre-incubated for 1 h with the inhibitor(s) in PBS, and the inhibitors were included in the DQ-gelatin/agarose/PBS mix. Inhibitor concentrations were 20 mM (EDTA), 1 mM (PMSF), 4.2 mM (Pefabloc-SC) and 2 µg/ml (aprotinin). Images were captured using a Leica DM4 microscope and LAS X software. For quantitative comparisons, identical conditions were used for image capture within each experiment. Quantitative analysis of the fractional area occupied by amyloid in fluorescence images of tissue sections stained with Congo red (as above) or Thioflavin S (ref. ^93^) was performed using the Fiji (ref. ^94^) implementation of ImageJ 1.52p.

### Statistics and reproducibility

Statistical analysis was performed using GraphPad Prism 5. Sample numbers reported relate to biological replicates, not technical replicates. Quantitative data were analysed by two-tailed Mann–Whitney test; categorical data were analysed by two-tailed Fisher’s exact test, with values of *P* < 0.05 being regarded as significant. Except where stated, all findings were replicated at least once in independent experiments.

### Reporting summary

Further information on research design is available in the Nature Research Reporting Summary linked to this article.

## Supplementary information

Supplementary information

Reporting summary

## Data Availability

The data supporting the findings from this study are available within the manuscript and its supplementary information. The mass spectrometry proteomics data have been deposited to the ProteomeXchange Consortium via the PRIDE partner repository^95^ under accession code PXD027747. Source data are provided with this paper.

## Source data

Source data

## References

[1] Benson MD (2020). Amyloid nomenclature 2020: update and recommendations by the international society of amyloidosis (ISA) nomenclature committee. Amyloid.

[2] Cornwell GG, Murdoch WL, Kyle RA, Westermark P, Pitkanen P (1983). Frequency and distribution of senile cardiovascular amyloid - a clinicopathologic correlation. Am. J. Med..

[3] Pitkanen P, Westermark P, Cornwell GG (1984). Senile systemic amyloidosis. Am. J. Pathol..

[4] Nakamichi K, Tachibana S (1998). Histology of the transverse carpal ligament and flexor tenosynovium in idiopathic carpal tunnel syndrome. J. Hand Surg. Am..

[5] Tanskanen M (2008). Senile systemic amyloidosis affects 25% of the very aged and associates with genetic variation in alpha2-macroglobulin and tau: a population-based autopsy study. Ann. Med..

[6] Sekijima Y (2011). High prevalence of wild-type transthyretin deposition in patients with idiopathic carpal tunnel syndrome: a common cause of carpal tunnel syndrome in the elderly. Hum. Pathol..

[7] Sueyoshi T (2011). Wild-type transthyretin-derived amyloidosis in various ligaments and tendons. Hum. Pathol..

[8] Treibel TA (2016). Occult transthyretin cardiac amyloid in severe calcific aortic stenosis: Prevalence and prognosis in patients undergoing surgical aortic valve replacement. Circ. Cardiovasc. Imaging.

[9] Longhi S (2016). Coexistence of degenerative aortic stenosis and wild-type transthyretin-related cardiac amyloidosis. JACC Cardiovasc. Imaging.

[10] Castano A (2017). Unveiling transthyretin cardiac amyloidosis and its predictors among elderly patients with severe aortic stenosis undergoing transcatheter aortic valve replacement. Eur. Heart J..

[11] Cavalcante JL (2017). Cardiac amyloidosis is prevalent in older patients with aortic stenosis and carries worse prognosis. J. Cardiovasc. Magn. Reson..

[12] Scully PR (2018). Prevalence of cardiac amyloidosis in patients referred for transcatheter aortic valve replacement. J. Am. Coll. Cardiol..

[13] Fontana M (2014). Native t1 mapping in transthyretin amyloidosis. JACC Cardiovasc. Imaging.

[14] Hutt DF (2014). Utility and limitations of 3,3-diphosphono-1,2-propanodicarboxylic acid scintigraphy in systemic amyloidosis. Eur. Heart J. Cardiovasc. Imaging.

[15] Perugini E (2005). Noninvasive etiologic diagnosis of cardiac amyloidosis using 99mtc-3,3-diphosphono-1,2-propanodicarboxylic acid scintigraphy. J. Am. Coll. Cardiol..

[16] Gillmore JD (2016). Nonbiopsy diagnosis of cardiac transthyretin amyloidosis. Circulation.

[17] Lane T (2019). Natural history, quality of life, and outcome in cardiac transthyretin amyloidosis. Circulation.

[18] Mohamed-Salem L (2018). Prevalence of wild type attr assessed as myocardial uptake in bone scan in the elderly population. Int. J. Cardiol..

[19] Rowczenio D (2019). Analysis of the ttr gene in the investigation of amyloidosis: A 25-year single uk center experience. Hum. Mutat..

[20] Jacobson DR (1997). Variant-sequence transthyretin (isoleucine 122) in late-onset cardiac amyloidosis in black americans. New Engl. J. Med..

[21] Wallace MR, Dwulet FE, Conneally PM, Benson MD (1986). Biochemical and molecular genetic characterization of a new variant prealbumin associated with hereditary amyloidosis. J. Clin. Invest..

[22] Sousa A, Coelho T, Barros J, Sequeiros J (1995). Genetic epidemiology of familial amyloidotic polyneuropathy (FAP)-type I in Povoa do Varzim and Vila do Conde (north of Portugal). Am. J. Med. Genet..

[23] Hellman U (2008). Heterogeneity of penetrance in familial amyloid polyneuropathy, attr val30met, in the swedish population. Amyloid.

[24] Gillmore JD, Hawkins PN (2015). V122I transthyretin variant in elderly black americans. New Engl. J. Med..

[25] Mangione PP (2014). Proteolytic cleavage of Ser52Pro variant transthyretin triggers its amyloid fibrillogenesis. Proc. Natl Acad. Sci. USA.

[26] Marcoux J (2015). A novel mechano-enzymatic cleavage mechanism underlies transthyretin amyloidogenesis. EMBO Mol. Med..

[27] Mangione PP (2018). Plasminogen activation triggers transthyretin amyloidogenesis in vitro. J. Biol. Chem..

[28] Pinkert CA, Ornitz DM, Brinster RL, Palmiter RD (1987). An albumin enhancer located 10 kb upstream functions along with its promoter to direct efficient, liver-specific expression in transgenic mice. Genes Dev..

[29] Reixach N (2008). Human-murine transthyretin heterotetramers are kinetically stable and non-amyloidogenic. A lesson in the generation of transgenic models of diseases involving oligomeric proteins. J. Biol. Chem..

[30] Zhao G (2008). Inconsistency between hepatic expression and serum concentration of transthyretin in mice humanized at the transthyretin locus. Genes Cells.

[31] Sato T (2012). STT3B-dependent posttranslational N-glycosylation as a surveillance system for secretory protein. Mol. Cell.

[32] Teixeira AC, Saraiva MJ (2013). Presence of n-glycosylated transthyretin in plasma of V30M carriers in familial amyloidotic polyneuropathy: an escape from erad. J. Cell. Mol. Med..

[33] Axelrad MA, Kisilevsky R, Willmer J, Chen SJ, Skinner M (1982). Further characterization of amyloid-enhancing factor. Lab Invest..

[34] Mollee, P. et al. Implementation and evaluation of amyloidosis subtyping by laser-capture microdissection and tandem mass spectrometry. *Clin. Proteomics* **13**, 10.1186/s12014-016-9133-x (2016). 10.1186/s12014-016-9133-x PMC5081679 27795698

[35] Dogan A (2017). Amyloidosis: Insights from Proteomics. Annual Review of Pathology: Mechanisms of Disease.

[36] Vrana JA (2009). Classification of amyloidosis by laser microdissection and mass spectrometry–based proteomic analysis in clinical biopsy specimens. Blood.

[37] Ihse E (2013). Amyloid fibrils containing fragmented ATTR may be the standard fibril composition in attr amyloidosis. Amyloid.

[38] Liz MA, Faro CJ, Saraiva MJ, Sousa MM (2004). Transthyretin, a new cryptic protease. J. Biol. Chem..

[39] Liz MA (2012). Transthyretin is a metallopeptidase with an inducible active site. Biochem. J..

[40] Raimondi S (2020). Comparative study of the stabilities of synthetic in vitro and natural ex vivo transthyretin amyloid fibrils. J. Biol. Chem..

[41] Lijnen HR, Okada K, Matsuo O, Collen D, Dewerchin M (1999). Alpha2-antiplasmin gene deficiency in mice is associated with enhanced fibrinolytic potential without overt bleeding. Blood.

[42] Collen D, Lijnen HR (1986). The fibrinolytic system in man. Crit. Rev. Oncol. Hematol..

[43] Rijken DC, Lijnen HR (2009). New insights into the molecular mechanisms of the fibrinolytic system. J. Thromb. Haemost..

[44] Tengborn L, Blomback M, Berntorp E (2015). Tranexamic acid - an old drug still going strong and making a revival. Thromb. Res..

[45] Ibrahim RB, Liu YT, Yeh SY, Tsai JW (2019). Contributions of animal models to the mechanisms and therapies of transthyretin amyloidosis. Front. Physiol..

[46] Sasaki H (1986). Generation of transgenic mice producing a human transthyretin variant: A possible mouse model for familial amyloidotic polyneuropathy. Biochem. Biophys. Res. Commun..

[47] Yi S (1991). Systemic amyloidosis in transgenic mice carrying the human mutant transthyretin (Met30) gene. Pathologic similarity to human familial amyloidotic polyneuropathy, type I. Am. J. Pathol..

[48] Kohno K (1997). Analysis of amyloid deposition in a transgenic mouse model of homozygous familial amyloidotic polyneuropathy. Am. J. Pathol..

[49] Takaoka Y (1997). Comparison of amyloid deposition in two lines of transgenic mouse that model familial amyloidotic polyneuropathy, type I. Transgenic Res..

[50] Teng MH (2001). Amyloid and nonfibrillar deposits in mice transgenic for wild-type human transthyretin: A possible model for senile systemic amyloidosis. Lab. Investig..

[51] Tagoe CE, Jacobson DR, Gallo G, Buxbaum JN (2003). Mice transgenic for human TTR have the same frequency of renal TTR deposition whether maintained in conventional or specific pathogen free environments. Amyloid.

[52] Tagoe CE, French D, Gallo G, Buxbaum JN (2004). Amyloidogenesis is neither accelerated nor enhanced by injections of preformed fibrils in mice transgenic for wild-type human transthyretin: The question of infectivity. Amyloid.

[53] Wei L (2004). Deposition of transthyretin amyloid is not accelerated by the same amyloid in vivo. Amyloid.

[54] Inoue S (2008). Specific pathogen free conditions prevent transthyretin amyloidosis in mouse models. Transgenic Res..

[55] Lipman RD, Gaillard ET, Harrison DE, Bronson RT (1993). Husbandry factors and the prevalence of age-related amyloidosis in mice. Lab Anim. Sci..

[56] Tagoe CE (2007). In vivo stabilization of mutant human transthyretin in transgenic mice. Amyloid.

[57] Matsumura A (1982). A novel amyloid fibril protein isolated from senescence-accelerated mice. Lab. Invest.

[58] Higuchi K, Naiki H, Kitagawa K, Hosokawa M, Takeda T (1991). Mouse senile amyloidosis. AS_sam_ amyloidosis in mice presents universally as a systemic age-associated amyloidosis. Virchows Arch. B Cell Pathol. Incl. Mol. Pathol..

[59] HogenEsch H (1993). Gastrointestinal AAPOAII and systemic AA-amyloidosis in aged C57BL/Ka mice. Amyloid-type dependent effect of long-term immunosuppressive treatment. Virchows Arch. B Cell Pathol. Incl. Mol. Pathol..

[60] Cowan AJ (2011). Macroglossia - not always AL amyloidosis. Amyloid.

[61] Obayashi K (2012). Pathological changes long after liver transplantation in a familial amyloidotic polyneuropathy patient. BMJ Case Rep.

[62] Oshima T (2014). Changes in pathological and biochemical findings of systemic tissue sites in familial amyloid polyneuropathy more than 10 years after liver transplantation. J. Neurol. Neurosurg. Psychiatry.

[63] Misumi Y (2014). Myopathic phenotype of familial amyloid polyneuropathy with a rare transthyretin variant: ATTR Ala45Asp. Amyloid.

[64] Ingenbleek Y, Bernstein LH (2015). Plasma transthyretin as a biomarker of lean body mass and catabolic states. Adv. Nutr..

[65] Baltz M. L., Caspi D., Hind C. R. K., Feinstein A. & Pepys M. B. *Amyloidosis* (eds Glenner G. G., Osserman E. F., Benditt E. P., Calkins E., Cohen A. S. & Zucker-Franklin D.) (Plenum Press, 1986).

[66] Jarrett JT, Berger EP, Lansbury PT (1993). The carboxy terminus of the beta amyloid protein is critical for the seeding of amyloid formation: implications for the pathogenesis of alzheimer’s disease. Biochemistry.

[67] Naiki H, Higuchi K, Nakakuki K, Takeda T (1991). Kinetic analysis of amyloid fibril polymerization in vitro. Lab Invest..

[68] Morozova-Roche LA (2000). Amyloid fibril formation and seeding by wild-type human lysozyme and its disease-related mutational variants. J. Struct. Biol..

[69] Saelices, L., et al. Amyloid seeding of transthyretin by ex vivo cardiac fibrils and its inhibition. *Proc Natl Acad Sci USA* **115**, E6741–50 (2018). 10.1073/pnas.1805131115 PMC6055172 29954863

[70] Yamada T (2020). Clinical characteristics and natural history of wild-type transthyretin amyloid cardiomyopathy in Japan. ESC Heart Fail..

[71] Heymans S (1999). Inhibition of plasminogen activators or matrix metalloproteinases prevents cardiac rupture but impairs therapeutic angiogenesis and causes cardiac failure. Nat. Med..

[72] Creemers E (2000). Disruption of the plasminogen gene in mice abolishes wound healing after myocardial infarction. Am. J. Pathol..

[73] Lluis F (2001). Urokinase-dependent plasminogen activation is required for efficient skeletal muscle regeneration in vivo. Blood.

[74] Suelves M (2002). Plasmin activity is required for myogenesis in vitro and skeletal muscle regeneration in vivo. Blood.

[75] Carmeliet P (1997). Urokinase but not tissue plasminogen activator mediates arterial neointima formation in mice. Circ. Res..

[76] Carmeliet P (1998). Receptor-independent role of urokinase-type plasminogen activator in pericellular plasmin and matrix metalloproteinase proteolysis during vascular wound healing in mice. J. Cell Biol..

[77] Carmeliet P, Moons L, Ploplis V, Plow E, Collen D (1997). Impaired arterial neointima formation in mice with disruption of the plasminogen gene. J. Clin. Invest..

[78] Heymans S (2005). Loss or inhibition of uPA or MMP-9 attenuates LV remodeling and dysfunction after acute pressure overload in mice. Am. J. Pathol..

[79] Gupta KK, Donahue DL, Sandoval-Cooper MJ, Castellino FJ, Ploplis VA (2017). Plasminogen activator inhibitor-1 protects mice against cardiac fibrosis by inhibiting urokinase-type plasminogen activator-mediated plasminogen activation. Sci. Rep..

[80] Moriwaki H, Stempien-Otero A, Kremen M, Cozen AE, Dichek DA (2004). Overexpression of urokinase by macrophages or deficiency of plasminogen activator inhibitor type 1 causes cardiac fibrosis in mice. Circ. Res..

[81] Stempien-Otero A (2006). Mechanisms of cardiac fibrosis induced by urokinase plasminogen activator. J. Biol. Chem..

[82] Levi M (2001). Deficiency of urokinase-type plasminogen activator-mediated plasmin generation impairs vascular remodeling during hypoxia-induced pulmonary hypertension in mice. Circulation.

[83] Gardsvoll H, Ploug M (2007). Mapping of the vitronectin-binding site on the urokinase receptor: Involvement of a coherent receptor interface consisting of residues from both domain I and the flanking interdomain linker region. J. Biol. Chem..

[84] Huai Q (2008). Crystal structures of two human vitronectin, urokinase and urokinase receptor complexes. Nat. Struct. Mol. Biol..

[85] Eugen-Olsen J (2010). Circulating soluble urokinase plasminogen activator receptor predicts cancer, cardiovascular disease, diabetes and mortality in the general population. J. Intern. Med..

[86] Zheng L, Baumann U, Reymond JL (2004). An efficient one-step site-directed and site-saturation mutagenesis protocol. Nucleic Acids Res..

[87] Gibson DG (2009). Enzymatic assembly of DNA molecules up to several hundred kilobases. Nat. Methods.

[88] Episkopou V (1993). Disruption of the transthyretin gene results in mice with depressed levels of plasma retinol and thyroid hormone. Proc. Natl Acad. Sci. USA.

[89] Laurell CB (1972). Electroimmuno assay. Scand. J. Clin. Lab Invest. Suppl..

[90] Puchtler H, Sweat F, Levine M (1962). On the binding of Congo red by amyloid. J. Histochem. Cytochem..

[91] Mangione PP (2017). Increasing the accuracy of proteomic typing by decellularisation of amyloid tissue biopsies. J. Proteom..

[92] Frederiks WM, Mook OR (2004). Metabolic mapping of proteinase activity with emphasis on in situ zymography of gelatinases: review and protocols. J. Histochem. Cytochem..

[93] Westermark GT, Johnson KH, Westermark P (1999). Staining methods for identification of amyloid in tissue. Methods Enzymol..

[94] Schindelin J (2012). Fiji: an open-source platform for biological-image analysis. Nat. Methods.

[95] Perez-Riverol Y (2019). The pride database and related tools and resources in 2019: Improving support for quantification data. Nucleic Acids Res..

[96] Canetti D (2020). Diagnostic amyloid proteomics: experience of the UK national amyloidosis centre. Clin. Chem. Lab. Med..

