# Supplementary material for: Plasmin activity promotes amyloid deposition in a transgenic model of human transthyretin amyloidosis
Source: Nat Commun. 2021 Dec 7;12:7112. doi: 10.1038/s41467-021-27416-z (PMC8651690; doi:10.1038/s41467-021-27416-z)
Supplement: Supplementary file 1 — Supplementary information [file 41467_2021_27416_MOESM1_ESM.pdf]

Supplementary information

Plasmin activity promotes amyloid deposition in a transgenic model of human transthyretin amyloidosis

Ivana Slamova, Rozita Adib, Stephan Ellmerich, Michal R. Golos, Janet A. Gilbertson, Nicola Botcher, Diana Canetti, Graham W. Taylor, Nigel Rendell, Glenys A. Tennent, Guglielmo Verona, Riccardo Porcari, P. Patrizia Mangione, Julian D. Gillmore, Mark B. Pepys, Vittorio Bellotti, Philip N. Hawkins, Raya Al-Shawi, J. Paul Simons

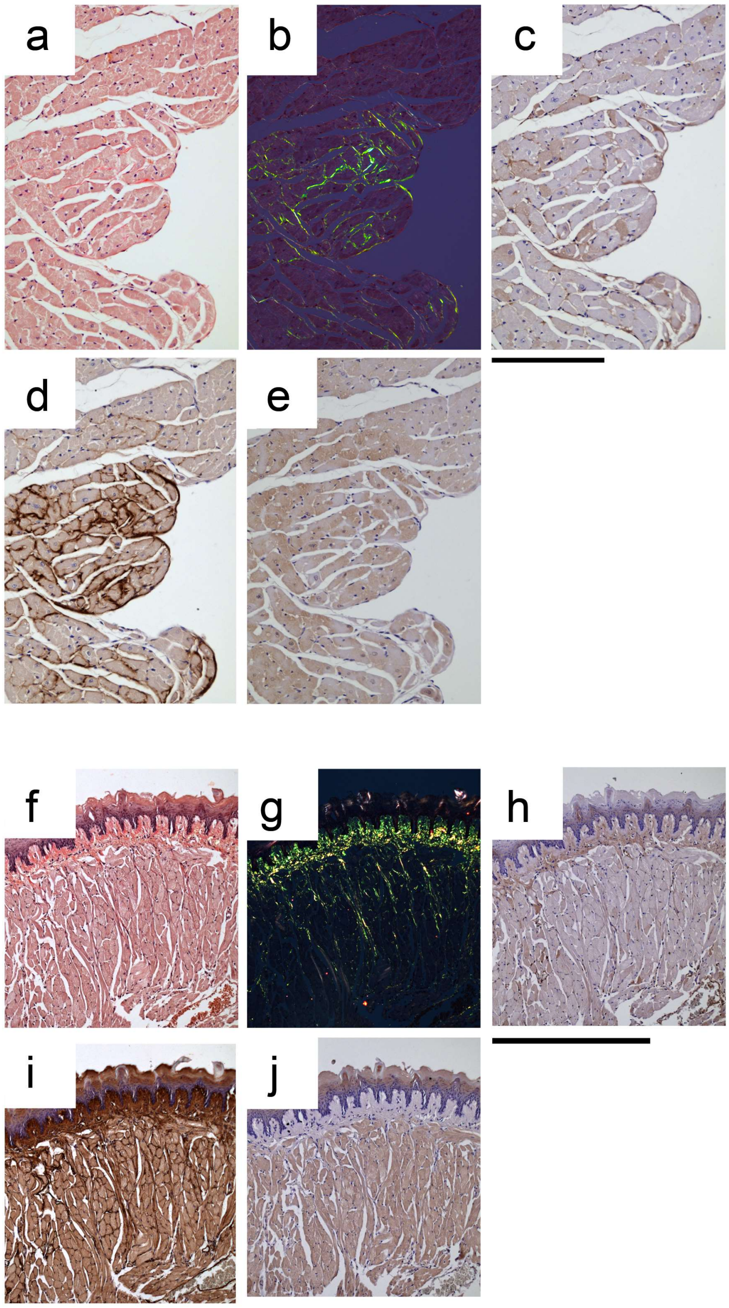

Supplementary Fig. 1 **Spontaneous AApoAII amyloidosis in a 24-month old mouse.**

Sections of heart (a-e) and tongue (f-j) of a 24-month old human TTR transgenic mouse were stained with Congo red (a, b, f, g) or detected by immunoperoxidase staining with antibodies to human TTR (c, h), mouse apoAII (d, i) or mouse SAA (e, j), and viewed under bright field (a, c-f, h-j) or cross polarised illumination (b, g). Amyloid was demonstrated by the pathognomonic red-green birefringence of Congo red stained extracellular deposits (a & b, f & g). The strong staining for apoAII of the amyloid deposits, identified by Congo red staining in adjacent sections, and the minimal staining for human TTR and mouse SAA, demonstrate clearly that amyloid fibril type in this mouse is apoAII. Of 59 unseeded mice analysed, this is the only one which developed amyloid. Scale bars: 100 µm (a-e) and 250 µm (f-j).

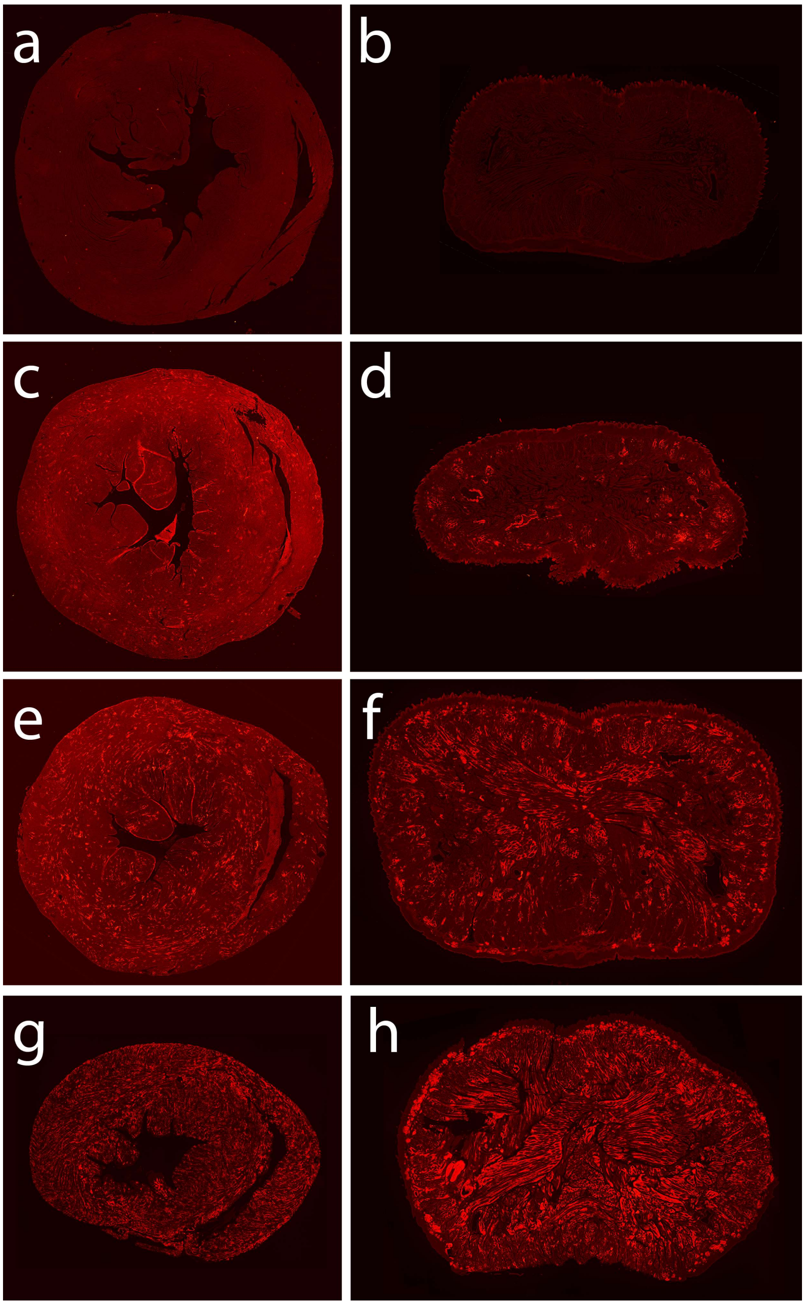

Supplementary Fig. 2 **Time course of amyloid deposition in hearts and tongues of line N4 male mice.**

The amount and extent of amyloid deposited in hearts (a,c,e,g) and tongues (b,d,e,f) of seeded mice increases progressively over months. Two months after seeding (a,b), very scattered deposits containing very small amounts of amyloid are visible (not obvious in this low-power view). Analysis after 3 months (c,d), 4 months (e,f) and 6 months (g,h) shows progressive increase in the amount and distribution of amyloid deposition. Images are representative of n = 10, 20, 8 and 15 different mice (a & b, c & d, e & f and g & h, respectively). Congo red-stained sections of heart (a,c,e,g) and tongue (b,d,e,f) from male line N4 transgenic mice were imaged by fluorescence microscopy; overlapping images were assembled to produce these composite images of entire sections. The imaging conditions were adjusted in each case to avoid saturation.

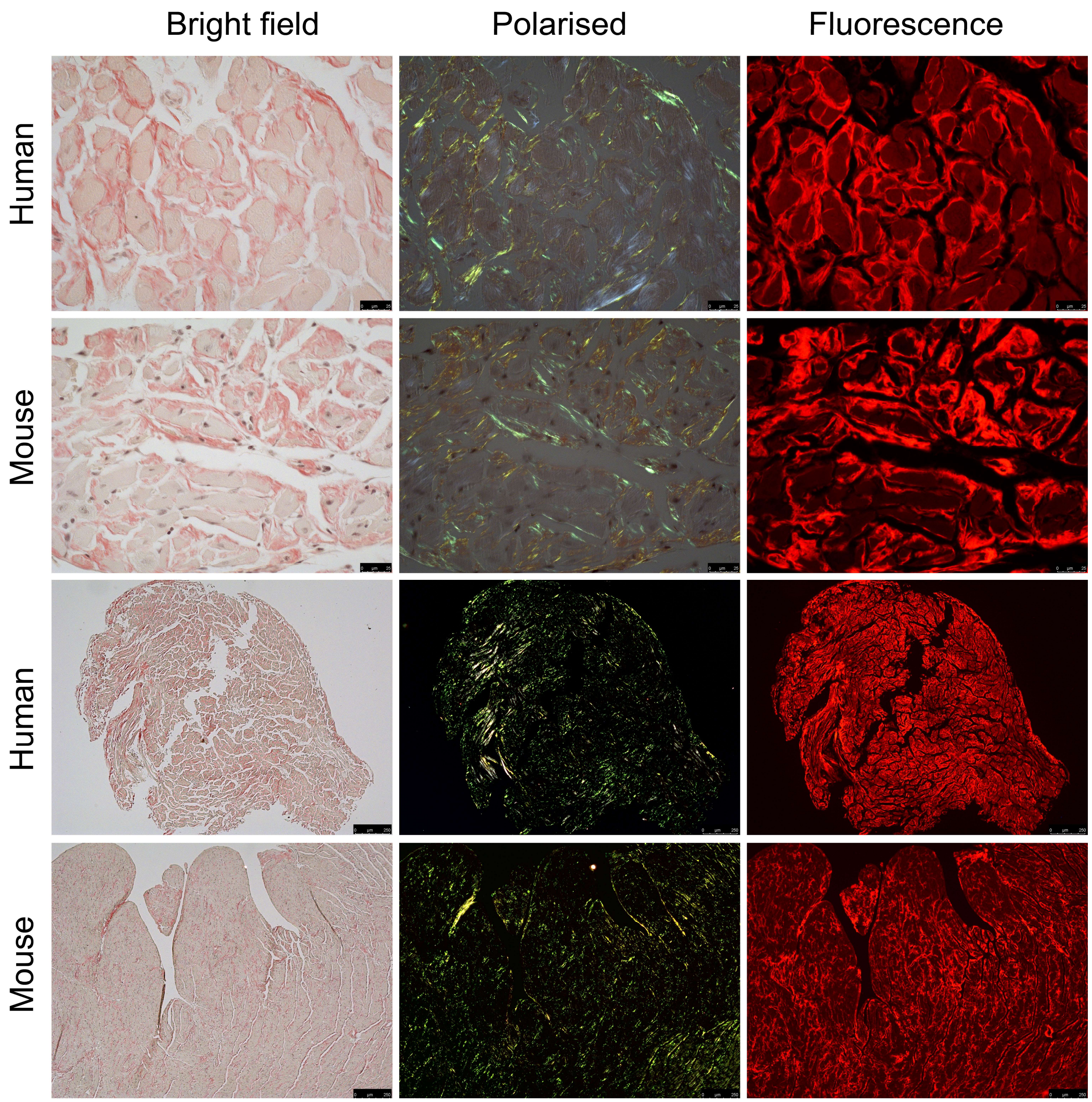

Supplementary Fig. 3 **Comparison of ATTR^{S52P} cardiac amyloid in a patient and a transgenic mouse.**

Congo red stained sections are shown, using bright field, polarising and fluorescence microscopy, as indicated. The human material was from an endomyocardial biopsy of a 30-year old male patient; the mouse tissue was from a female mouse and was collected 12 months after seeding. The low power images of the human biopsy show the full extent of the section.

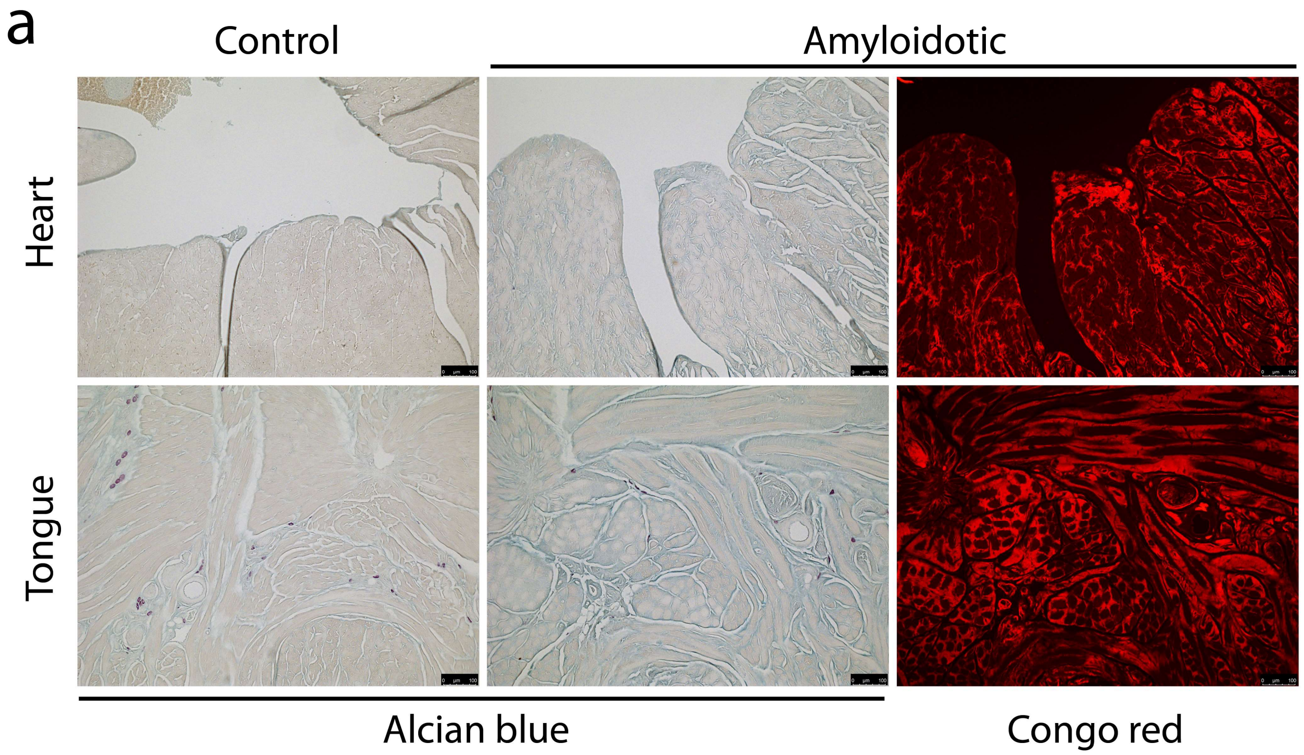

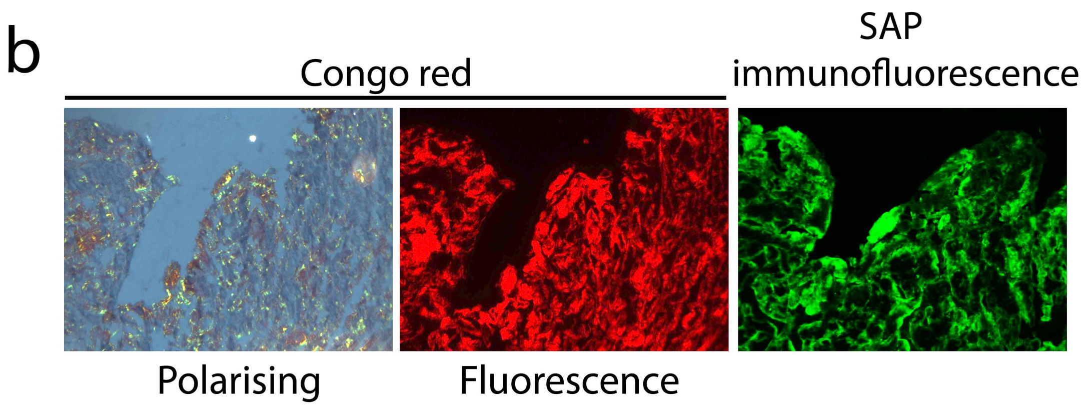

Supplementary Fig. 4 **Presence of proteoglycans and serum amyloid P component in mouse ATTR amyloid deposits.**

a) Sections of control and amyloidotic mouse heart and tongue tissue were stained, as indicated, with alcian blue to show the distribution of proteoglycans; representative of two mice analysed. The Congo red fluorescence images (of closely adjacent sections) show the distribution of the amyloid deposits.

b) Cryosections of an ATTR amyloidotic mouse heart are shown, stained with Congo red and imaged by polarising or fluorescence microscopy, or by immunofluorescence for mouse serum amyloid P component (SAP), as indicated.

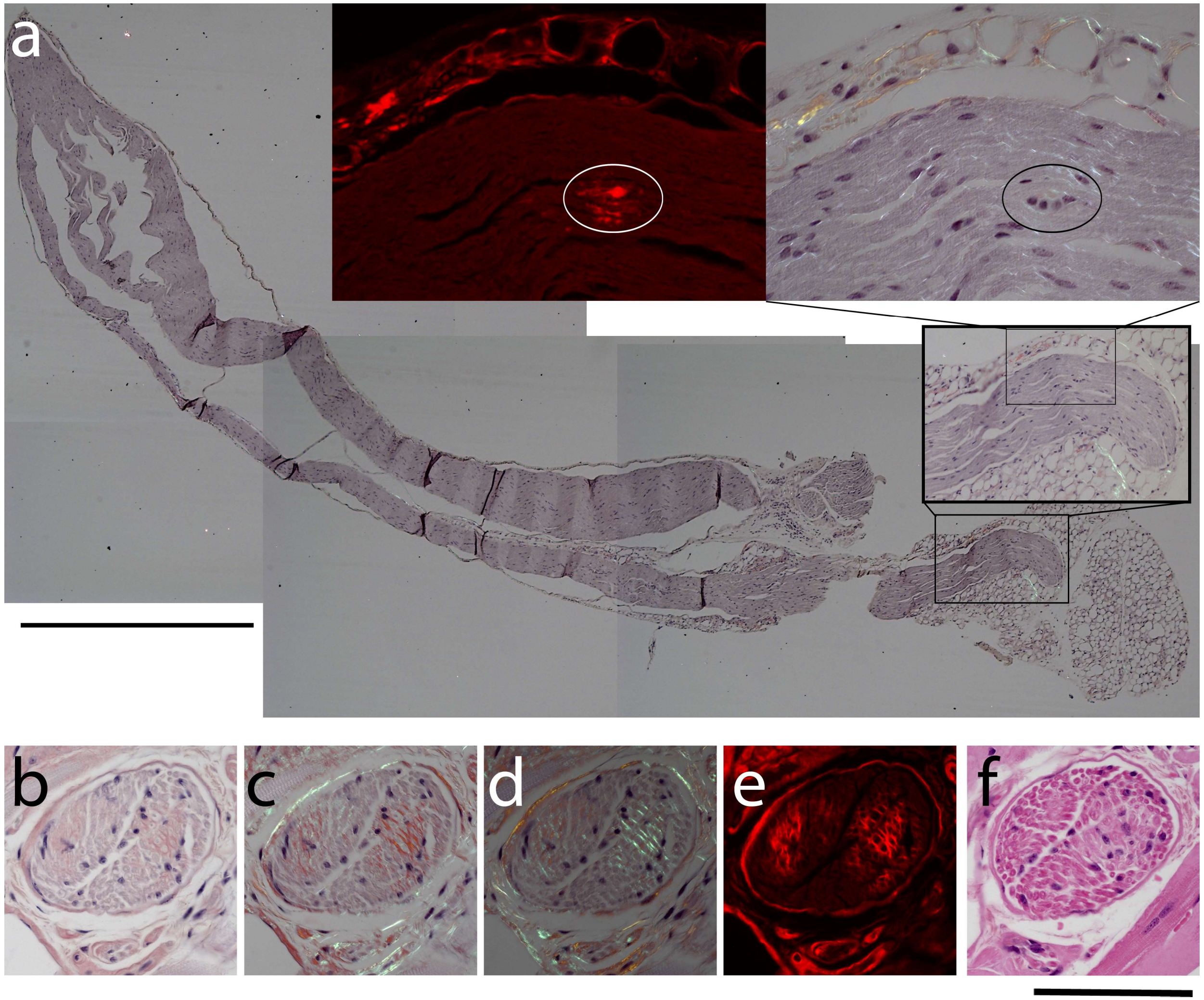

Supplementary Fig. 5 **Distribution of amyloid in other sites in line N4 mice - nerve**

Amyloid was detected within the sciatic nerve in only a single animal, 12 months after seeding, and only in a single small deposit (though amyloid was frequently found in epineurium). (a). The typical absence of amyloid within sciatic nerves contrasted with the abundant amyloid found in small nerve branches in the tongue from 6 months after seeding. b-e: Congo red stained section of tongue showing a nerve branch, imaged using bright field (b), polarising (c,d), or fluorescence (e) microscopy. f: H&E stained section, closely adjacent to that shown in b-e. Scale bars: a: 1 mm; b: 100 µm.

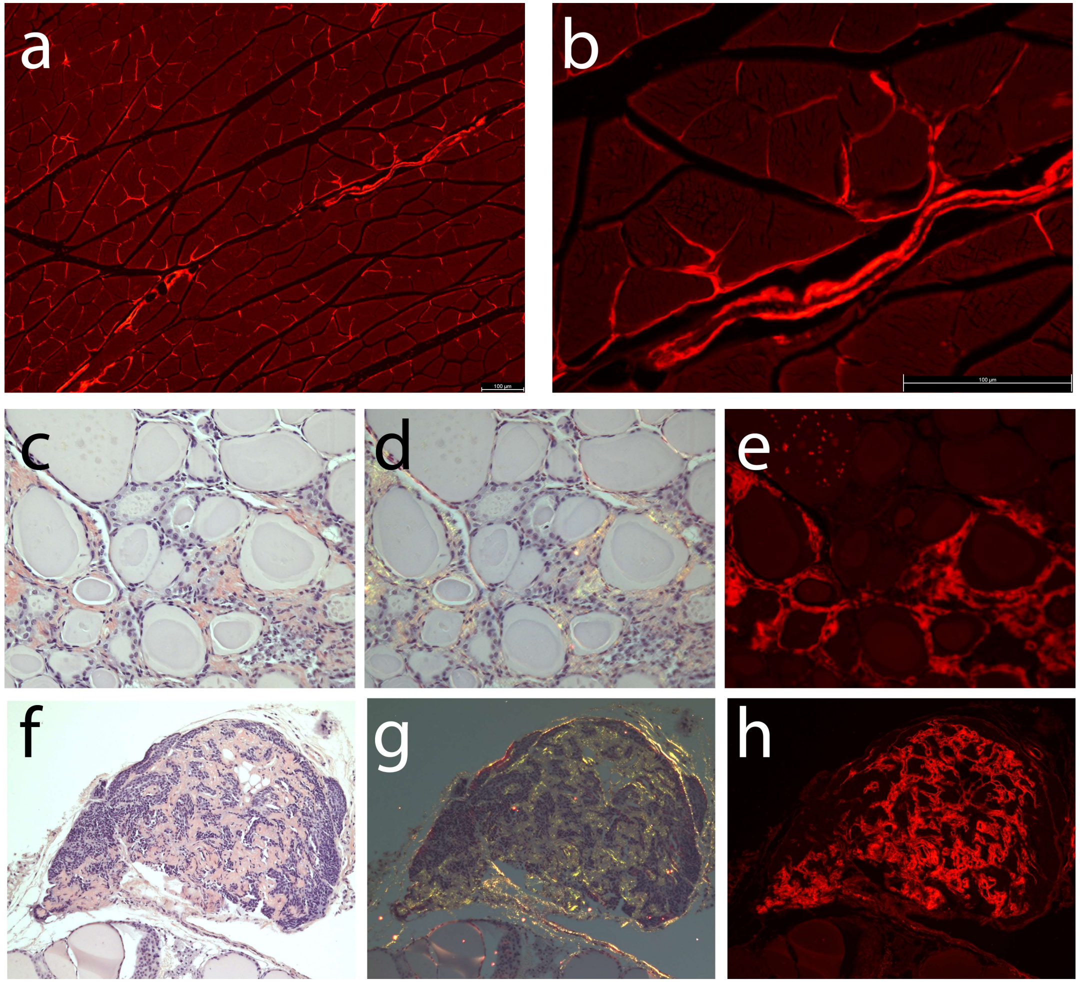

Supplementary Fig. 6 **Distribution of amyloid in other sites in line N4 mice – muscle, thyroid and parathyroid glands**

Congo red stained sections showing amyloid in (a, b): gastrocnemius muscle 12 months after seeding of a female mouse, (c – e): thyroid gland 6 months after seeding of a female mouse, and (f – h): parathyroid gland 6 months after seeding of a female mouse, imaged using fluorescence (a, b, e, h), bright field (c, f) or polarising (d, g) microscopy.

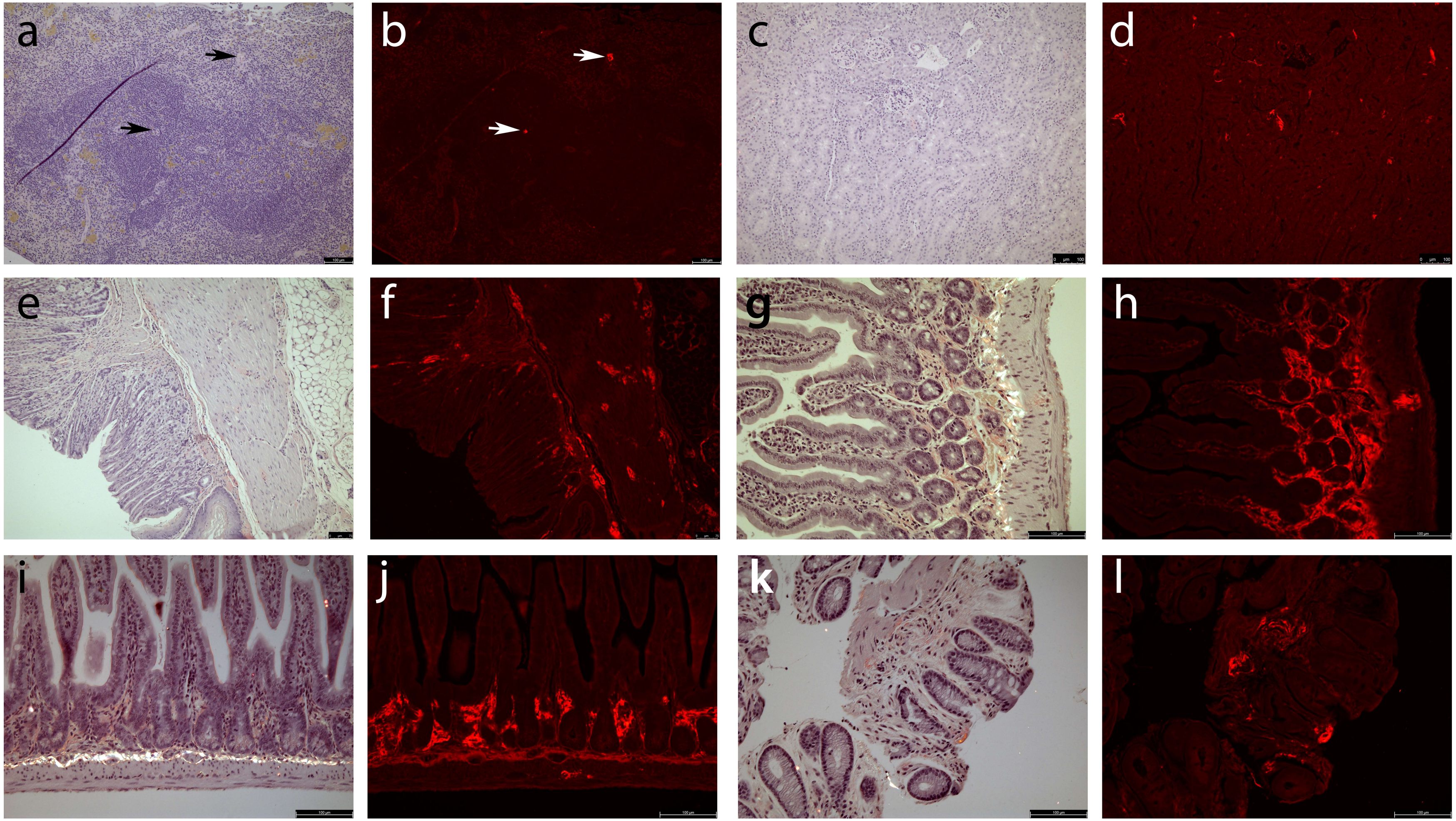

Supplementary Fig. 7 **Distribution of amyloid in other sites in line N4 mice – spleen, kidney and gastrointestinal tract**

Congo red stained sections showing (a, b): very small amyloid deposits in the spleen of a female mouse 12 months after seeding, (c, d): small amyloid deposits in the kidney of a female mouse 12 months after seeding and (e – l): amyloid in the gastrointestinal tract of a female mouse 6 months after seeding. e,f: stomach; g,h: duodenum; I,j: ileum; k,l: colon. Images were captured using bright field (a, c, e, g, i, k) or fluorescence microscopy (b, d, f, h, j, l)

a

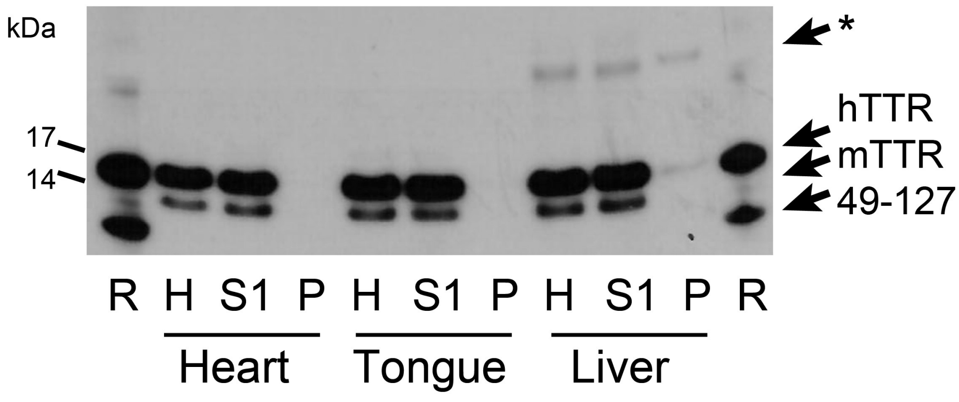

b

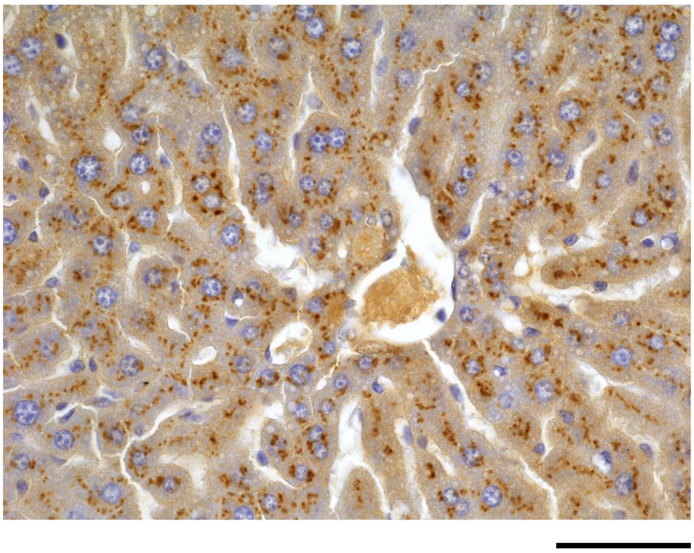

Supplementary Fig. 8 **Absence of insoluble human TTR in heart and tongue of unseeded human TTR^{S52P} transgenic mice but intracellular human TTR inclusions in hepatocytes.**

a) Extracts of the heart, tongue and liver of a 23 month-old line O5 transgenic male mouse were fractionated by sequential centrifugation and washing, and analysed by western blotting for human TTR; data representative of n=4 mice. H: unfractionated homogenate; S1: first supernatant; P: pellet (after 4th round of centrifugation); R: recombinant human TTR^{S52P} protein subjected to limited trypsin digestion; the glycosylated human TTR^{S52P} band is indicated by an asterisk. Source data are provided as a Source Data file.

b) Section of liver of a human TTR^{S52P} line O5 transgenic mouse stained by immunoperoxidase with anti-human TTR antibodies, showing discrete intracellular accumulations of the human protein, corresponding to the minor band of insoluble TTR in (a). Amongst 10 mice analysed, inclusions detectable by immunohistochemistry were variably present or absent and, when present, were not uniformly distributed in the tissue. Scale bar: 50 µm.

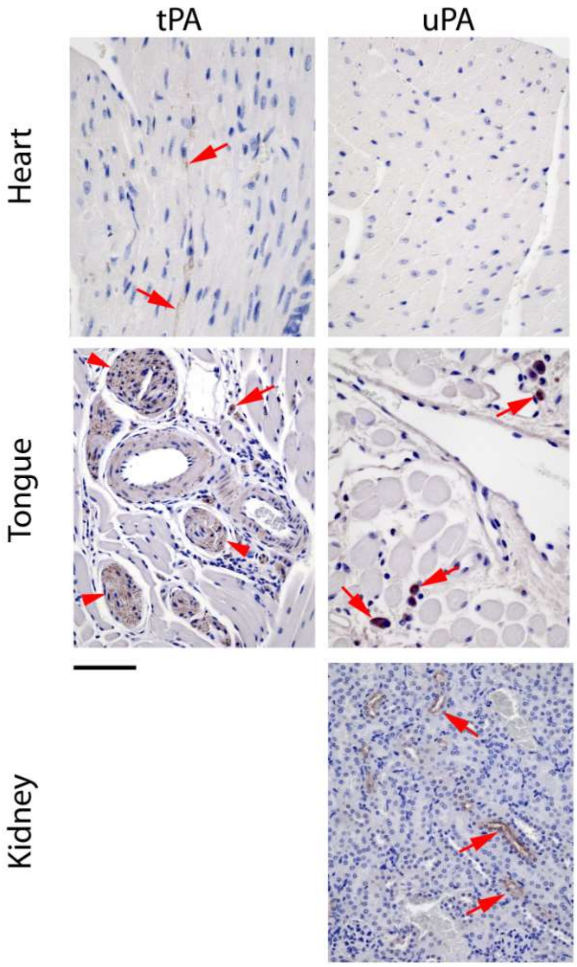

Supplementary Fig. 9 **tPA and uPA in control mouse tissues**

Immunohistochemical staining of non-amyloidotic mouse heart, tongue and renal cortex with anti-tPA and anti-uPA antisera, as indicated. In heart, endothelial staining for tPA (arrows) was observed in a minority of vessels, as previously reported [1]; there was no clear positive signal when staining normal heart tissue for uPA. In tongue, staining for tPA reveals signal in nerves (arrowheads) and mast cells (arrows), which are both documented sites of tPA expression [2,3]. Staining for uPA in the tongue was limited to mast cells (arrows), consistent with known expression in these cells [4]. Immunostaining of kidney gave signal in tubular epithelial cells (arrows), characteristic of uPA [5,6]. The images are representative of 2 biological replicates (heart and tongue) and 3 biological replicates (kidney).

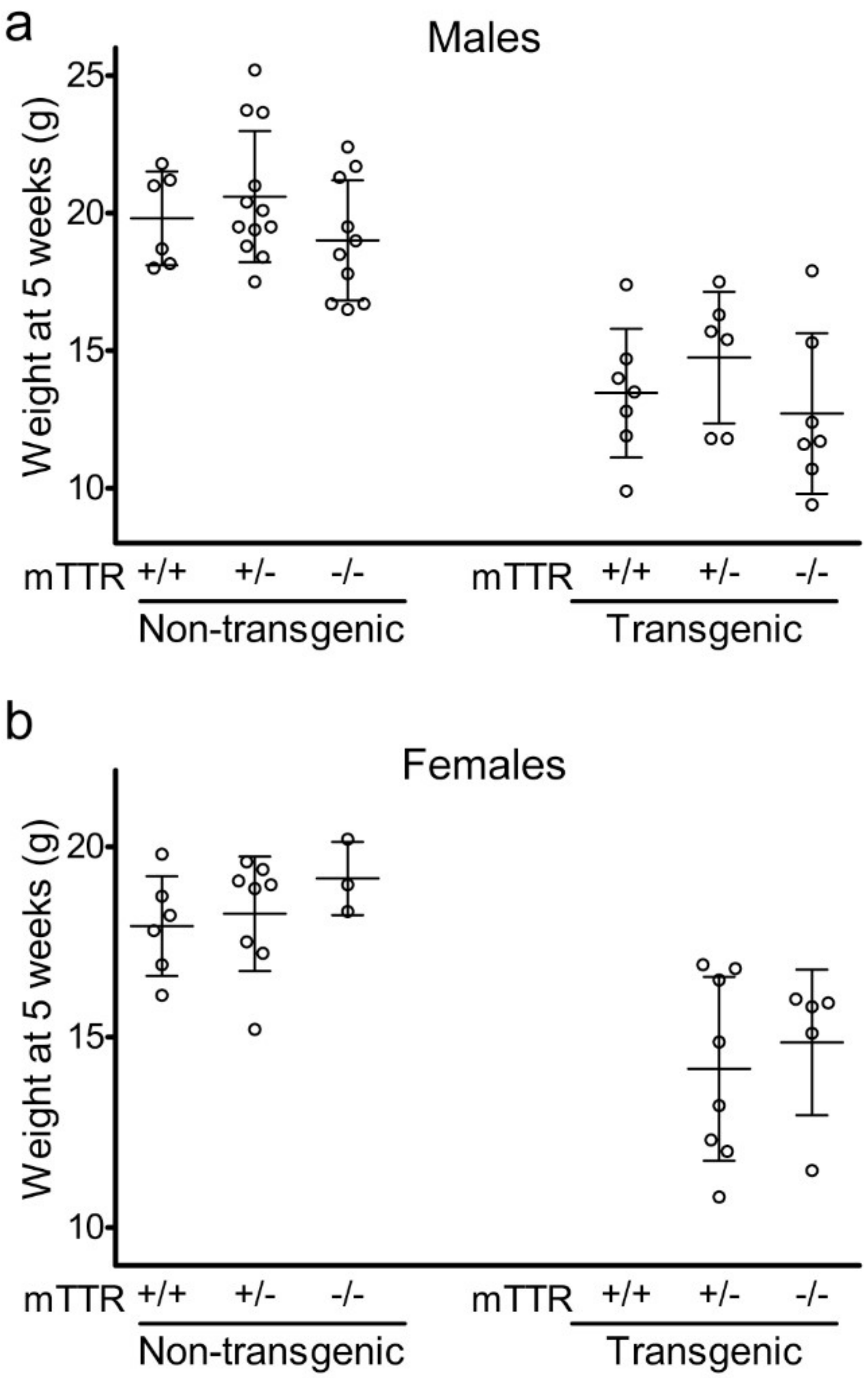

Supplementary Fig. 10 **Effect of genotype on body weight in line O5 mice.**

The weights (values, mean & SD) at 5 weeks of age of individual male and female mice of the different genotypes are shown in a & b respectively. In males, human TTR transgene genotype had a significant effect on weight (P<0.0001; F=77.07, 1 df), but endogenous mouse TTR genotype did not (P=0.101; F=2.422, 2 df). There was no interaction between mouse TTR and human TTR genotypes with respect to weight (P=0.949; F=0.05, 2 df); 2-way ANOVA. Insufficient data were collected from females for analysis by 2-way ANOVA. Nevertheless, transgenic females were also significantly smaller than non-transgenics (considering mouse $TTR^{+/-}$ females alone, P=0.0011; pooling females of the different mouse TTR genotypes, P<0.0001, 2-tailed Mann-Whitney tests). Source data are provided as a Source Data file.

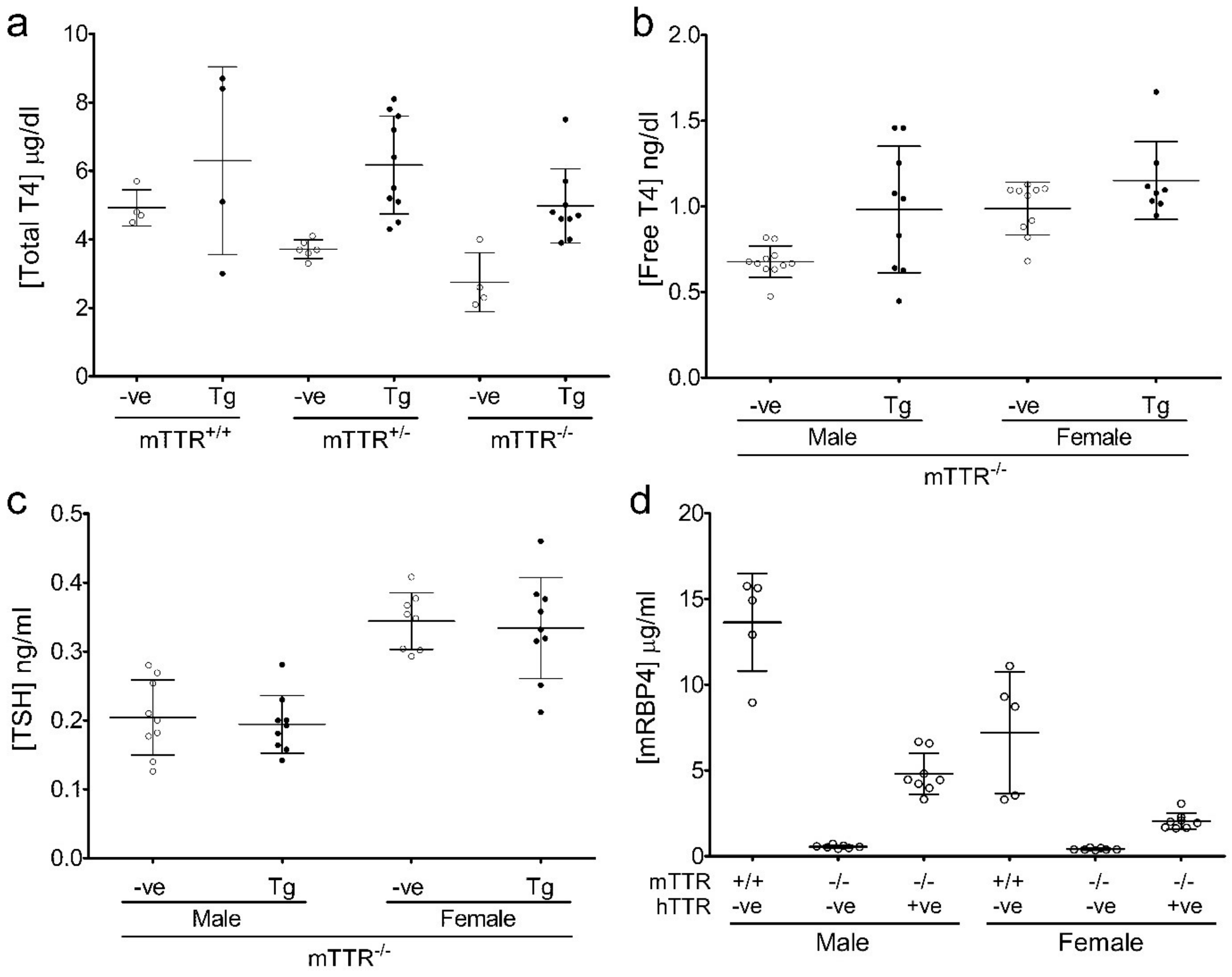

Supplementary Fig. 11 **Effects of genotype on thyroid hormone status and circulating retinol binding protein concentration in line O5 mice.**

Assayed serum concentrations (values for individual mice, mean & SD) of total T4, free T4, TSH and RBP4 are shown in panels a, b, c & d, respectively, with mouse genotypes indicated. The data were analysed by 2-way ANOVA. (a) Total T4 concentrations were significantly influenced by human TTR genotype (*P*=0.00014; F=18.78, 1 df) and by mouse TTR genotype (*P*=0.0209; F=4.39, 2 df). (b) Free T4 concentrations were significantly affected by human TTR genotype (*P*=0.0033; F=9.97, 1 df) and by sex (*P*=0.0027; F=10.47, 1 df). (c) While TSH concentration was significantly influenced by sex (*P*<0.0001; F=57.01, 1 df), there was no such effect of the human TTR transgene (*P*=0.5927; F=0.2922, 1 df). (d) RBP4 concentrations were significantly affected by TTR genotype (*P*<0.0001; F=105.8, 2df) and by sex (*P*<0.0001; F=33.56, 1 df). As previously reported, mice lacking TTR are profoundly deficient for RBP4 [7]. The expression of human TTR^{S52P} in $mTTR^{-/-}$ mice partially rescued RBP4 concentrations. RBP4 concentrations were higher in male transgenic mice than in females, corresponding to the higher TTR concentrations in males. Source data are provided as a Source Data file.

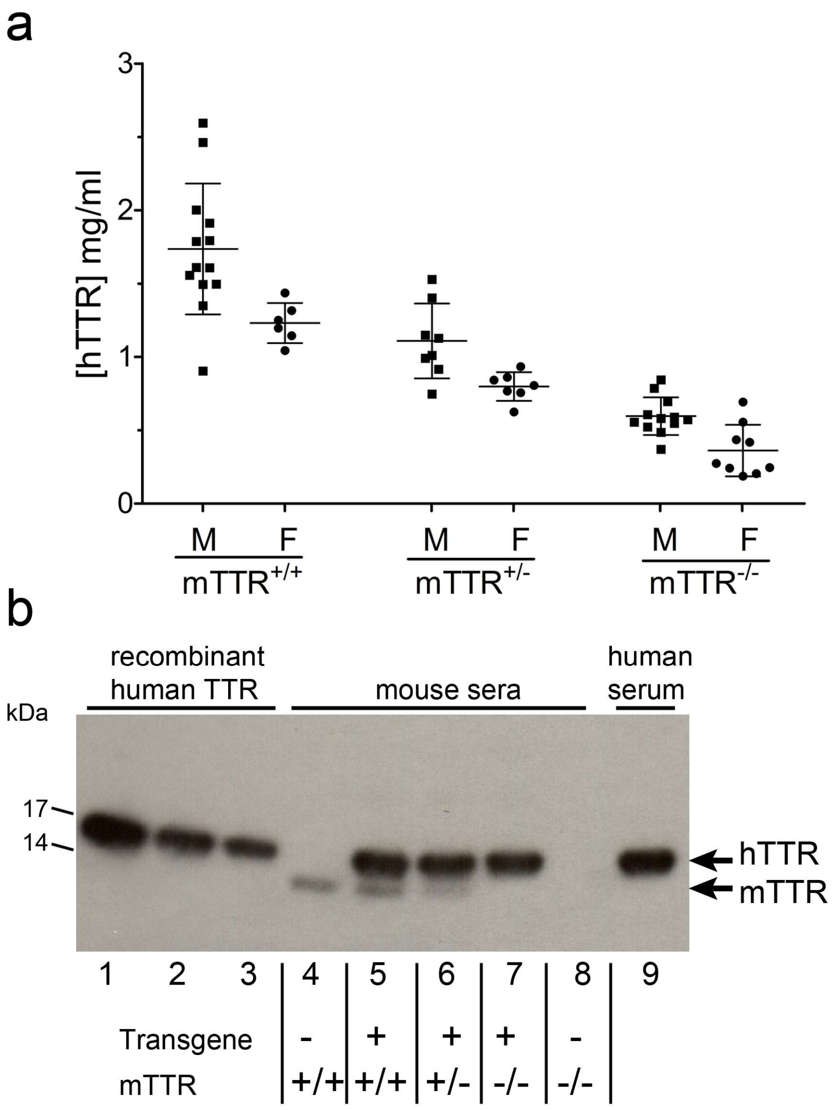

Supplementary Fig. 12 **Human TTR protein in the circulation of line O5 hTTR^{S52P} transgenic mice.**

(a) Human TTR concentrations measured in the sera of line O5 transgenic mice were measured by electroimmunoassay calibrated with isolated pure human wild-type TTR; non-transgenic mice gave no signal in this assay. Results (individual values, mean & SD) are presented for male (M) and female (F) mice that were wild-type for the endogenous mouse *ttr* gene ($ttr^{+/+}$), heterozygous ($ttr^{+/-}$) or homozygous $ttr^{-/-}$) for a mouse TTR null allele (n = 13, 8, and 26 for $ttr^{+/+}$, $ttr^{+/-}$, $ttr^{-/-}$ male mice, respectively, and n = 6, 7 and 24 for $ttr^{+/+}$, $ttr^{+/-}$, $ttr^{-/-}$ female mice, respectively). Analysis by 2-way ANOVA showed that the measured concentrations were greater in males than in females (P<0.0001; F= 22.34, 1 df) and were significantly influenced by mouse TTR genotype (P<0.0001; F= 65.94, 1 df). Only the results in mouse TTR null animals provide actual concentrations of human TTR. (b) Western blot of transgenic mouse sera. Under the conditions used, the mouse TTR protomer migrates slightly ahead of the human TTR protomer. Lanes 1, 2 and 3 contained 200, 100 and 50 ng, respectively, of recombinant human TTR, mixed with mouse TTR knockout serum. Lanes 4 – 8: sera of mice of different genotypes, as indicated. Lane 9, human serum. Each lane contained 0.2 µl serum. Source data are provided as a Source Data file.

	No. amyloidotic / Total no. of mice										
	Females			Males							totals
Time after seeding (months)	4	7	11	4	5	6	7	8	10	11	
mTTR$^{+/+}$		2/2	1/2	2/2			2/2	1/1	1/1	2/2	11/12
mTTR$^{+/-}$	0/1		0/2				3/3			2/2	5/8
mTTR$^{-/-}$	0/2	1/3	1/2	2/4	5/5	2/2	5/6	3/3		4/4	23/31
All mTTR genotypes	0/3	3/5	2/6	4/6	5/5	2/2	10/11	4/4	1/1	8/8	39/51

Supplementary Table 1 **Summary of amyloid deposition in line O5 mice**

Prevalence of genuine Congophilic amyloid deposits in the tissues of line O5 human TTR^{S52P} transgenic mice that had received parenteral injection of human TTR^{S52P} amyloidotic spleen homogenate 4 to 11 months previously, summarised according to time, mouse TTR genotype and sex.

	No. of mice with amyloid / total no. of mice			
Line & sex	O5 male		N1 female	
Months after seeding	4	7	4	7
Amyloid (cardiac and/or lingual)	6 / 8	8 / 8	1 / 8	8 / 9
Cardiac amyloid	5 / 8	7 / 8	0 / 8	7 / 9
Lingual amyloid	6 / 8	8 / 8	1 / 8	8 / 9

Supplementary Table 2 **TTR concentration-independent effect of sex on TTR amyloid deposition**

Comparison of amyloid deposition in line O5 males and line N1 females. Despite the higher concentration of TTR in line N1 females than line O5 males (*P*=0.0005, Mann-Whitney test), a greater proportion of seeded line O5 males than line N1 females contained amyloid when analysed 4 months after seeding with homogenate of human TTR^{S52P} amyloidotic spleen (*P*=0.041, Fisher's exact test). This experiment was not repeated.

Supplementary references

1. Levin EG, Santell L, Osborn KG. The expression of endothelial tissue plasminogen activator in vivo: A function defined by vessel size and anatomic location. *J Cell Sci* **110 (Pt 2)**, 139-148 (1997).
2. Sillalber C*, et al.* The mast cell as site of tissue-type plasminogen activator expression and fibrinolysis. *Journal of Immunology* **162**, 1032-1041 (1999).
3. Siconolfi LB, Seeds NW. Mice lacking tpa, upa, or plasminogen genes showed delayed functional recovery after sciatic nerve crush. *J Neurosci* **21**, 4348-4355 (2001).
4. Dwyer DF, Barrett NA, Austen KF, Immunological Genome Project C. Expression profiling of constitutive mast cells reveals a unique identity within the immune system. *Nat Immunol* **17**, 878-887 (2016).
5. Wagner SN, Atkinson MJ, Wagner C, Hofler H, Schmitt M, Wilhelm O. Sites of urokinase-type plasminogen activator expression and distribution of its receptor in the normal human kidney. *Histochem Cell Biol* **105**, 53-60 (1996).
6. Sappino AP, Huarte J, Vassalli JD, Belin D. Sites of synthesis of urokinase and tissue-type plasminogen activators in the murine kidney. *J Clin Invest* **87**, 962-970 (1991).
7. Wei S*, et al.* Studies on the metabolism of retinol and retinol-binding protein in transthyretin-deficient mice produced by homologous recombination. *J Biol Chem* **270**, 866-870 (1995).
