# Supplementary material for: Plasmin activity promotes amyloid deposition in a transgenic model of human transthyretin amyloidosis
Source: Nat Commun. 2021 Dec 7;12:7112. doi: 10.1038/s41467-021-27416-z (PMC8651690; doi:10.1038/s41467-021-27416-z)
Supplement: Supplementary file 2 — Reporting summary [file 41467_2021_27416_MOESM2_ESM.pdf]

nature portfolio

Corresponding author(s): Paul Simons

Last updated by author(s): Nov 8, 2021

Reporting Summary

Nature Portfolio wishes to improve the reproducibility of the work that we publish. This form provides structure for consistency and transparency in reporting. For further information on Nature Portfolio policies, see our Editorial Policies and the Editorial Policy Checklist.

Statistics

For all statistical analyses, confirm that the following items are present in the figure legend, table legend, main text, or Methods section.

n/a	Confirmed	
☐	☒	The exact sample size (*n*) for each experimental group/condition, given as a discrete number and unit of measurement
☐	☒	A statement on whether measurements were taken from distinct samples or whether the same sample was measured repeatedly
☐	☒	The statistical test(s) used AND whether they are one- or two-sided<br>*Only common tests should be described solely by name; describe more complex techniques in the Methods section.*
☐	☒	A description of all covariates tested
☒	☐	A description of any assumptions or corrections, such as tests of normality and adjustment for multiple comparisons
☐	☒	A full description of the statistical parameters including central tendency (e.g. means) or other basic estimates (e.g. regression coefficient) AND variation (e.g. standard deviation) or associated estimates of uncertainty (e.g. confidence intervals)
☐	☒	For null hypothesis testing, the test statistic (e.g. *F*, *t*, *r*) with confidence intervals, effect sizes, degrees of freedom and *P* value noted<br>*Give P values as exact values whenever suitable.*
☒	☐	For Bayesian analysis, information on the choice of priors and Markov chain Monte Carlo settings
☒	☐	For hierarchical and complex designs, identification of the appropriate level for tests and full reporting of outcomes
☒	☐	Estimates of effect sizes (e.g. Cohen's *d*, Pearson's *r*), indicating how they were calculated

Our web collection on statistics for biologists contains articles on many of the points above.

Software and code

Policy information about availability of computer code

Data collection	Microscope images were captured using Leica Application Suite X
Data analysis	Graphpad Prism 5 was used for statistical analyses. Fiji implementation of Image J 1.52p was used for image analysis.

For manuscripts utilizing custom algorithms or software that are central to the research but not yet described in published literature, software must be made available to editors and reviewers. We strongly encourage code deposition in a community repository (e.g. GitHub). See the Nature Portfolio guidelines for submitting code & software for further information.

Data

Policy information about availability of data

All manuscripts must include a data availability statement. This statement should provide the following information, where applicable:

- Accession codes, unique identifiers, or web links for publicly available datasets
- A description of any restrictions on data availability
- For clinical datasets or third party data, please ensure that the statement adheres to our policy

The data supporting the findings from this study are available within the manuscript and its supplementary information. The mass spectrometry proteomics data have been deposited to the ProteomeXchange Consortium via the PRIDE partner repository under accession code PXD027747 [https://www.ebi.ac.uk/pride/archive/projects/PXD027747]. Source data are provided with this paper.

Field-specific reporting

Please select the one below that is the best fit for your research. If you are not sure, read the appropriate sections before making your selection.

- [x] Life sciences
- [ ] Behavioural & social sciences
- [ ] Ecological, evolutionary & environmental sciences

For a reference copy of the document with all sections, see nature.com/documents/nr-reporting-summary-flat.pdf

Life sciences study design

All studies must disclose on these points even when the disclosure is negative.

Sample size	For exploratory analyses, sample sizes of 1 or 2 were used. For many experiments the sample sizes were influenced by random factors beyond our control (including breeding performance, random segregation of transgenes and random deviations from 1:1 sex ratio), and to avoid wastage. Choices of sample sizes were informed by power calculations using the program G*power and previous experience of the of amyloid induction experiments (e.g. Simons et al (2013) PNAS 110:16115-16120), in which there can be considerable variability in the timing and extent of amyloid deposition.
Data exclusions	No data were excluded except for one experiment in which amyloid deposition was being compared in groups of male and female line N4 mice which were heterozygous or homozygous for α2-antiplasmin knockout. There was insufficient amyloid in the female mice of either group for quantitative analysis, so the two female groups were excluded from analysis.
Replication	All findings were replicated at least once in independent experiments except for (1) deglycosylation of human TTR(S52P) shown in Fig 1b, the purpose of which was to confirm the correct identification of the glycosylated TTR band and which gave unequivocal results in technical replicates, and (2) comparison of amyloid deposition in line O5 males and line N1 females which was not repeated because of availability of mice.
Randomization	Allocation to groups was semi-random. Full randomisation was constrained because of group housing of mice.
Blinding	Where appropriate (i.e. for comparison of different conditions), analyses were blinded. Blinding was not appropriate for other analyses (e.g. description of the pattern of amyloid deposition), and blinding was impossible for some analyses (e.g. comparison of histological findings in amyloidotic and non-amyloidotic mice, because sample identities were clearly apparent from their appearance).

Reporting for specific materials, systems and methods

We require information from authors about some types of materials, experimental systems and methods used in many studies. Here, indicate whether each material, system or method listed is relevant to your study. If you are not sure if a list item applies to your research, read the appropriate section before selecting a response.

Materials & experimental systems

n/a	Involved in the study
[ ]	[x] Antibodies
[x]	[ ] Eukaryotic cell lines
[x]	[ ] Palaeontology and archaeology
[ ]	[x] Animals and other organisms
[ ]	[x] Human research participants
[x]	[ ] Clinical data
[x]	[ ] Dual use research of concern

Methods

n/a	Involved in the study
[x]	[ ] ChIP-seq
[x]	[ ] Flow cytometry
[x]	[ ] MRI-based neuroimaging

Antibodies

Antibodies used	TTR Western blotting: Sheep anti-human prealbumin - Affinity purified; The Binding Site Product code AU066.X Batch 190102. Detected with Polyclonal Rabbit Anti-Sheep Immunoglobulins-HRP; Dako product code P0163<br>TTR immunohistochemistry: rabbit anti-human TTR purified immunoglobulin; Dako product code A0002. Detected with ImmPRESS HRP Horse Anti-Rabbit IgG Polymer Detection Kit; Vector laboratories catalogue number MP-7401<br>SAA immunohistochemistry: goat anti-mouse Serum amyloid A1/A2 antibody; R&D Systems catalogue number AF2948. Detected with ImmPRESS HRP Horse Anti-Goat IgG Polymer Detection Kit, Peroxidase; Vector laboratories catalogue number MP-7405<br>ApoAII immunohistochemistry: rabbit anti-mouse ApoAII antiserum; gift from Prof. Keiichi Higuchi, Shinshu University, Japan. Detected with ImmPRESS HRP Horse Anti-Rabbit IgG Polymer Detection Kit; Vector laboratories catalogue number MP-7401<br>Plasminogen Western blotting: Rabbit anti mouse plasminogen, antigen affinity purified; Molecular Innovations product code ASMPLG-GF-HT, Lot number ASMPLG-GF-HT-613. Detected with polyclonal Goat Anti-Rabbit Immunoglobulins-HRP; Dako product code P00048<br>Plasminogen immunohistochemistry: Rabbit anti mouse plasminogen, antigen affinity purified; Molecular Innovations product code ASMPLG-GF-HT, Lot number ASMPLG-GF-HT-613. Detected with ImmPRESS HRP Horse Anti-Rabbit IgG Polymer Detection Kit; Vector laboratories catalogue number MP-7401

	Urokinase Western blotting: Mouse monoclonal mouse anti-uPA/urokinase/PLAU Antibody (H77A10); Santa Cruz Biotechnology catalogue number sc-59727. Detected with Clean-Blot™ IP Detection Reagent (HRP); Thermo-Fisher catalogue number 21230<br>Urokinase immunohistochemistry: Rabbit anti-mouse uPA, antigen affinity purified; Molecular Innovations product code ASMUPA-GF-HT, lot number ASMUPA-GF-HT-619. Detected with ImmPRESS HRP Horse Anti-Rabbit IgG Polymer Detection Kit; Vector laboratories catalogue number MP-7401<br>GAPDH Western blotting: Rabbit monoclonal anti-GAPDH (clone 14C10). Cell Signaling Technology catalogue number 2118S. Detected with polyclonal Goat Anti-Rabbit Immunoglobulins-HRP; Dako product code P00048.<br>tPA immunohistochemistry: Rabbit anti mouse tPA, antigen affinity purified; Molecular Innovations product code ASMTPA-GF-HT, lot number ASMTPA-GF-HT-914. Detected with ImmPRESS HRP Horse Anti-Rabbit IgG Polymer Detection Kit; Vector laboratories catalogue number MP-7401
Validation	TTR western blotting: antibody manufacturer validated as monospecific by immunoelectrophoresis, and as TTR-reactive by double diffusion against pure human TTR and human serum. In-house validation: by Western blotting, the antibody recognises purified recombinant TTR and the 49-127 tryptic fragment generated in vitro. No signal was detected by Western blotting of TTR knockout mouse samples, mouse was TTR detected in wild-type mouse samples and human TTR was recognised in human TTR-expressing transgenic mouse serum and tissue samples, with signal proportional to human TTR content measured by independent methods.<br>TTR immunohistochemistry - manufacturer validated for in vitro diagnostic use; mono-specific by crossed immunoelectrophoresis. In-house validation: antibody recognises ATTR amyloid in human patients, and does not cross-react with known mouse AA amyloid or mouse AApoAII amyloid.<br>SAA immunohistochemistry: antibody manufacturer validated by Western blotting with mouse serum and recombinant mouse SAA. In-house validation: recognises AA amyloid by immunohistochemistry, but not ATTR or AApoAII amyloid.<br>ApoAII antibody: reported to be specific by Prof. Higuchi. In-house validation: recognises AApoAII amyloid by immunohistochemistry, but not ATTR or AA amyloid.<br>Plasminogen: manufacturer-validated for Western blotting and immunohistochemistry. In-house validation: recognises pure mouse plasminogen, and gives bands of expected sizes for plasminogen and plasmin in mouse serum and tissue homogenates.<br>uPA polyclonal Ab: manufacturer-validated for immunohistochemistry. In-house validation: gives expected staining pattern in renal tubules and mast cells in control tissues<br>uPA monoclonal Ab: manufacturer validated for Western blotting . In-house validation: gives bands of expected sizes in mouse tissue samples.<br>GAPDH: manufacturer validated for Western blotting . In-house validation: gives bands of expected sizes in mouse tissue samples.<br>tPA polyclonal Ab: manufacturer-validated for immunohistochemistry. In-house validation: gives expected staining pattern in endothelial cells, mast cells and nerves in control tissues

Animals and other organisms

Policy information about studies involving animals; ARRIVE guidelines recommended for reporting animal research

Laboratory animals	Mouse, transgenic for Albumin enhancer/promoter-human TTRS52P transgene in combination with mouse TTR knockout or α2-antiplasmin (Serpinf2) knockout on C57BL/6 background. Line O5 TTR transgenic mice were generated and maintained on the C57BL/6 background; lines N1 and N4 were generated on a mixed C57BL/6 x CBA background and backcrossed onto C57BL/6 and Knockout alleles were originally generated in 129/SvJ stem cells and were backcrossed onto C57BL/6. Both sexes used, up to two years of age.
Wild animals	None
Field-collected samples	None
Ethics oversight	All animal studies were ethically reviewed by the UCL Royal Free Campus Animal Welfare and Ethical Review Body and carried out in accordance with the provisions of the United Kingdom Animals (Scientific Procedures) Act 1986 and local rules.

Note that full information on the approval of the study protocol must also be provided in the manuscript.

Human research participants

Policy information about studies involving human research participants

Population characteristics	Single patient with familial ATTR amyloidosis
Recruitment	Archival post-mortem tissue (donated with informed written consent, in accordance with the Helsinki Declaration).
Ethics oversight	Ethics Committee of the Royal Free Hampstead NHS Trust

Note that full information on the approval of the study protocol must also be provided in the manuscript.
